# Unraveling the Simultaneous Enhancement of Selectivity and Durability on Single‐Crystalline Gold Particles for Electrochemical CO_2_ Reduction

**DOI:** 10.1002/advs.202201491

**Published:** 2022-05-02

**Authors:** Yun Ji Lim, Dongho Seo, Syed Asad Abbas, Haeun Jung, Ahyeon Ma, Kug‐Seung Lee, Gaehang Lee, Hosik Lee, Ki Min Nam

**Affiliations:** ^1^ Department of Chemistry and Chemistry Institute for Functional Materials Pusan National University Geumjeong‐gu Busan 46241 Republic of Korea; ^2^ 8C Nano Probe XAFS Beamline Pohang Accelerator Laboratory Pohang 37673 Republic of Korea; ^3^ Korea Basic Science Institute (KBSI) Daejeon 34133 Republic of Korea; ^4^ Department of Energy Engineering School of Energy and Chemical Engineering Ulsan National Institute of Science and Technology (UNIST) Ulsan 44919 Republic of Korea

**Keywords:** electrochemical CO_2_ reduction, polydiallyldimethylammonium chloride, single‐crystalline gold, surface functionalization

## Abstract

Electrochemical carbon dioxide reduction is a mild and eco‐friendly approach for CO_2_ mitigation and producing value‐added products. For selective electrochemical CO_2_ reduction, single‐crystalline Au particles (octahedron, truncated‐octahedron, and sphere) are synthesized by consecutive growth and chemical etching using a polydiallyldimethylammonium chloride (polyDDA) surfactant, and are surface‐functionalized. Monodisperse, single‐crystalline Au nanoparticles provide an ideal platform for evaluating the Au surface as a CO_2_reduction catalyst. The polyDDA‐Au cathode affords high catalytic activity for CO production, with >90% Faradaic efficiency over a wide potential range between −0.4 and −1.0 V versus RHE, along with high durability owing to the consecutive interaction between dimethylammonium and chloride on the Au surface. The influence of polyDDA on the Au particles, and the origins of the enhanced selectivity and stability are fully investigated using theoretical studies. Chemically adsorbed polyDDA is consecutively affected the initial adsorption of CO_2_ and the stability of the *CO_2_, *COOH, and *CO intermediates during continuous CO_2_ reduction reaction. The polyDDA functionalization is extended to improving the CO Faradaic efficiency of other metal catalysts such as Ag and Zn, indicating its broad applicability for CO_2_ reduction.

## Graphical abstract

Single‐crystalline Au provides an ideal platform for evaluating the Au surface as a CO_2_ reduction catalyst. The polyDDA functionalized Au electrode shows high catalytic activity and durability for CO production. Interestingly, polyDDA functionalization can be widely applied to other metal catalysts such as Ag and Zn. This functionalization affords broad applicability for utilizing metal catalysts for efficient CO_2_ reduction reactions.

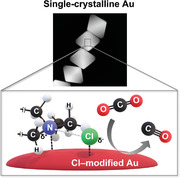

## 1 Introduction

The continuous consumption of fossil fuels has resulted in the overproduction of CO_2_, leading to rapid global warming and climate change.^[^ ^1^, ^2^, ^3^, ^4^, ^5^ ^]^ The electrochemical CO_2_ reduction reaction (CO_2_RR) is a promising solution for utilizing this excessive CO_2_.^[^ ^6^ ^]^ The catalytic CO_2_RR under mild conditions is an eco‐friendly approach not only for reducing excess CO_2_, but also for producing valuable fuels that can be applied in renewable energy utilization.^[^ ^7^, ^8^, ^9^, ^10^ ^]^ Because CO_2_ is a thermodynamically stable molecule, an adequate catalyst and electrolyte with high external energy are needed to facilitate the electrochemical reduction reaction.^[^ ^11^ ^]^ Both the hydrogen evolution reaction (HER) and CO_2_RR consume protons (H^+^) and electrons, where the HER competes with the selective CO_2_RR. Typically, when a large overpotential is applied during the electrochemical reaction, the HER is promoted, which reduces the Faradaic efficiency for the CO_2_RR.^[^ ^12^ ^]^

Metallic electrodes are promising candidates as CO_2_RR catalysts for CO production.^[^ ^13^, ^14^, ^15^, ^16^, ^17^ ^]^ Particularly, Au nanoparticles have attracted considerable interest as CO_2_RR catalysts because of their relatively high CO Faradaic efficiency.^[^ ^18^ ^]^ Although Au nanoparticles are benchmark catalysts in terms of activity, their sustainability for selective CO production remains a major challenge. There are many papers that are efficient in a narrow potential range, but it is not easy to show good Faradaic efficiency in a wide potential range. The size and exposed surface facets of the Au catalysts can play a critical role in the further development of the CO_2_RR.^[^ ^19^ ^]^ Au nanoparticles of different shapes, such as cubes, needles, rods, rhomboids, dodecahedrons, and those having porous structures have been investigated.^[^ ^20^, ^21^, ^22^, ^23^ ^]^ Most Au nanoparticles have a large surface area and thus afford a high current density; however, these small nanoparticles generally preferentially promote the undesired HER.^[^ ^24^ ^]^ Density functional theory (DFT) calculations indicate that the edge sites of the Au catalyst promote CO formation, whereas corner sites accelerate the HER.^[^ ^25^ ^]^ Small Au nanoparticles are easily deactivated in the CO_2_RR due to surface coalescence during the electrocatalytic reaction, which reduces the catalyst stability.^[^ ^26^ ^]^ Because the activity and stability arise from conflicting features, identifying a proper catalyst that is both active and stable for the CO_2_RR remains a challenge.

Surface functionalization on the molecular level can accelerate the CO_2_RR and suppress the undesired HER.^[^ ^27^, ^28^ ^]^ For instance, the surface of Au nanoparticles has been modified with specific functional groups, such as chloride anions,^[^ ^29^ ^]^ *N*‐heterocyclic carbenes,^[^ ^30^ ^]^ and amines,^[^ ^31^ ^]^ which enhance the initial stability of major intermediates, leading to rapid CO formation.^[^ ^32^ ^]^ Typically, the functionalization of the surface of Au with Cl^−^ and CN^−^ species drastically enhances the CO_2_RR selectivity and activity compared with that of bare Au. However, functionalized Au showed poor stability due to the desorption of the adsorbed anions during the continuous CO_2_RR.^[^ ^29^ ^]^ Stabilizing specific functional groups on the surface of Au can develop into a new approach for improving the CO_2_RR performance in various metal electrodes.

Herein, we report the synthesis and surface functionalization of single‐crystalline Au particles for sustainable electrochemical CO_2_RR. Single‐crystalline Au particles are synthesized by a consecutive process of growth and chemical etching in the presence of polydiallyldimethylammonium chloride (polyDDA) as a surfactant. The prepared polyDDA‐Au particles have a monodispersed size distribution and are single‐crystalline, making them an ideal system for studying the catalytic properties of the metal surface for the CO_2_RR. As a cathode, the polyDDA‐Au particles afford high catalytic activity, with over 90% Faradaic efficiency of CO production over a wide potential range. The influence of polyDDA on the Au particles, and the origins of the enhanced selectivity and stability are fully investigated using theoretical studies. Based on the results, polyDDA functionalization is applied to other metal catalysts such as silver (Ag) and zinc (Zn), which also exhibit highly improved CO selectivity compared with that of the untreated counterparts, indicating the broad applicability of polyDDA in the utilization of metal catalysts for sustainable CO_2_ reduction.

## 2 Result and Discussion

### 2.1 Synthesis of Uniform and polyDDA‐Functionalized Single‐Crystalline Au Particles

**Figure 1:**
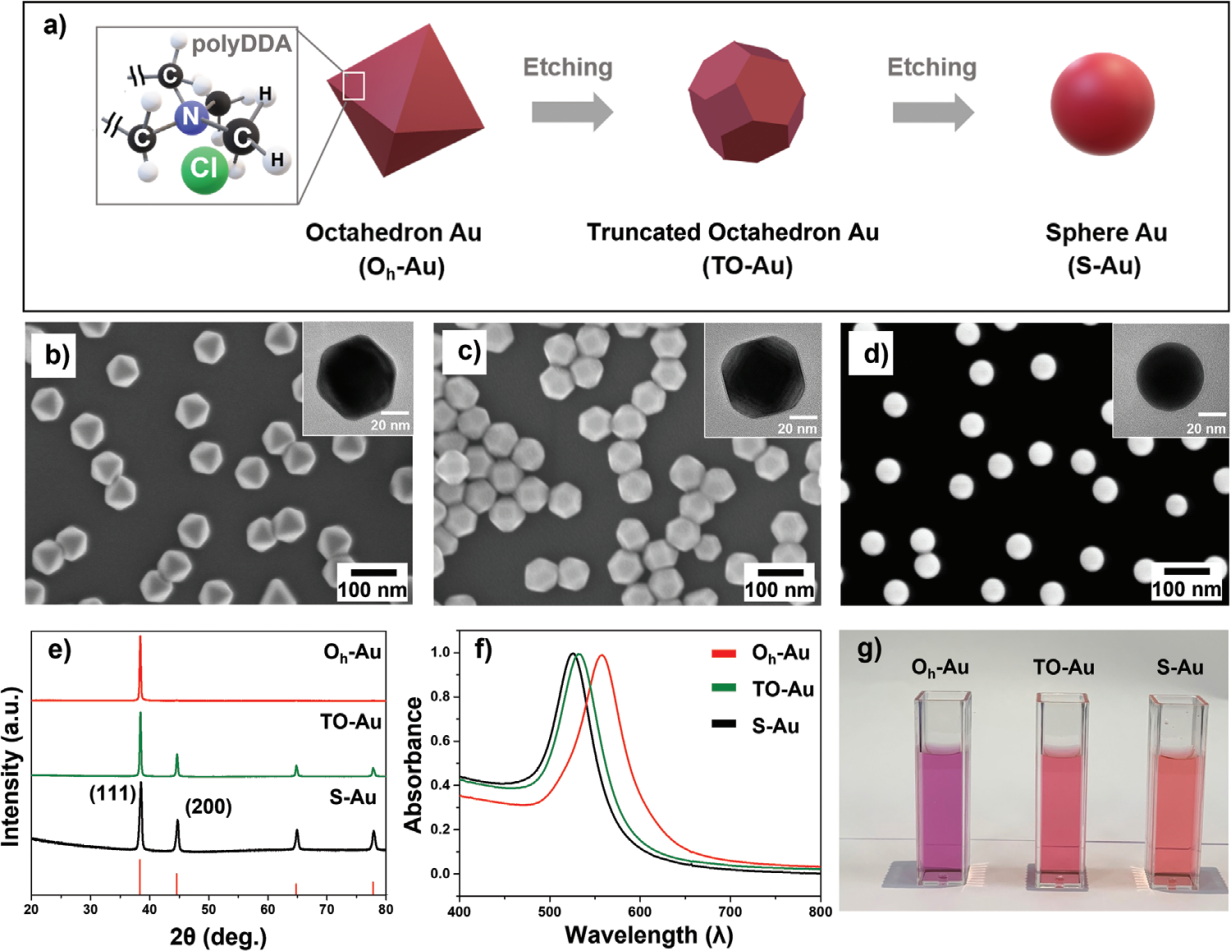
Characterization of polyDDA‐modified octahedron (O_h_), truncated‐octahedron (TO), and spherical (S) Au particles. a) Schematic of synthesis of polyDDA‐Au nanoparticles. SEM and TEM (inset) images of b) O_h_‐Au, c) TO‐Au, and d) S‐Au particles. e) XRD patterns, f) UV–vis absorbance, and g) photographic image of O_h_‐Au, TO‐Au, and S‐Au particles.

Single‐crystalline Au particles were synthesized by a polyol process, by modifying a literature method (**Figure** **1**a).^[^ ^33^ ^]^ Octahedral (O_h_‐Au) particles were formed by reducing the Au precursor (HAuCl_4_) in the presence of the polyDDA surfactant. By varying the molar ratio of the Au precursor and polyDDA, O_h_‐Au particles of various sizes (35–150 nm) were obtained (Figure S1, Supporting Information).

Figure 1b shows scanning electron microscopy (SEM) and transmission electron microscopy (TEM, inset) images of the O_h_‐Au nanoparticles, demonstrating the uniform size of the octahedral particles, with an average edge length of 65.4 ± 2.2 nm. The high‐resolution TEM and corresponding fast Fourier‐transform (FFT) data indicate that the Au octahedron was single‐crystalline with a (111) exposed plane (Figure S2, Supporting Information). The surface composition of the O_h_‐Au particles was investigated using X‐ray photoelectron spectroscopy (XPS) (Figure S3, Supporting Information). The XPS profile showed Au 4f peaks with typical N and Cl peaks, indicating that polyDDA was bound to the O_h_‐Au surface. To control the exposed surface facets, the O_h_‐Au particles were chemically etched using HAuCl_4_ as the initial Au precursor (Figure 1a).^[^ ^34^ ^]^ The O_h_‐Au particles started to dissolve at the vertices and edges and produced uniform truncated octahedral Au (TO‐Au) particles after 24 h of etching (Figure 1c and Figure S2, Supporting Information). Further chemical etching produced spherical Au (S‐Au) particles with an average diameter of 53.5 ± 1.5 nm (Figure 1d). The TEM and corresponding FFT images indicated that the TO‐Au and S‐Au particles were also single‐crystalline (Figure S2, Supporting Information). Note that the size of the Au particles gradually decreased during the etching process. Although the shape of the Au particles changed during chemical etching, polyDDA was still bound to the TO‐Au and S‐Au surfaces, as indicated by the XPS data (Figure S3, Supporting Information). To further investigate the coordination environment of Au in the presence of polyDDA, X‐ray absorption near edge structure (XANES) analysis of O_h_‐Au was performed (Figure S4, Supporting Information). The X‐ray absorption near edge structure (XANES) showed Au‐L_3_ absorption edge at 11 919.0 eV, which is comparable to 11 918.6 eV for Au foil (Figure S4a, Supporting Information). The Au‐L_3_ absorption spectra correspond to the transition of 2p_3/2_ electrons to empty states in 5d_5/2_ and 5d_3/2_ levels where most of the d‐band states are occupied. The O_h_‐Au and Au foil absorption edges are very close but a small shift which might be attributed to Au—Cl bond.^[^ ^35^ ^]^ The extended X‐ray absorption fine structure (EXAFS) in the R profile exhibited a characteristic peak position consistent with Au—Cl coordination at 2.0 Å (Figure S4b, Supporting Information)^[^ ^36^ ^]^ along with Au—Au peak at 2.98 Å.

The crystal structure of the Au nanoparticles was investigated using X‐ray diffraction (XRD) (Figure 1e). All XRD peaks well matched with those of the face‐centered cubic Au phase (JCPDS No. 04–0784). The UV–vis spectra of the Au particles were acquired in the range of 400–800 nm to further characterize the size‐ and shape‐dependent properties (Figure 1f,g). The surface plasmon resonance extinction profile of the O_h_‐Au particle solution showed a single resonance at 558 nm, indicating that the edge length was ≈60 nm (Figure 1f),^[^ ^37^ ^]^ which is in good agreement with the SEM image (Figure 1b). The extinction peaks of the TO‐Au and S‐Au particles were observed at 531 and 525 nm (Figure 1f), from which the particle diameter was determined to be ≈50 nm.^[^ ^38^ ^]^ The extinction maximum was progressively blue‐shifted as the Au particle size decreased, and an additional shift was observed owing to the spherical shape of the Au particles.^[^ ^39^ ^]^ Figure S5, Supporting Information, shows the thermogravimetric analysis (TGA) curve of the O_h_‐Au particles that were surface‐functionalized with polyDDA. The small initial weight loss (≈0.1%) at 220 °C corresponds to the desorption of water on the Au surface. The mass reduction (≈1.0%) observed at 220–500 °C corresponds to the decomposition of the polyDDA capping layer, which indicates that the Au surface was coated with 1.0 wt% polyDDA. The prepared polyDDA‐functionalized Au particles were monodispersed and single‐crystalline, providing an ideal system for investigating the catalytic properties of the Au surface for the CO_2_RR.

### 2.2 Electrochemical CO_2_ Reduction on polyDDA‐Functionalized Au Particles

**Figure 2:**
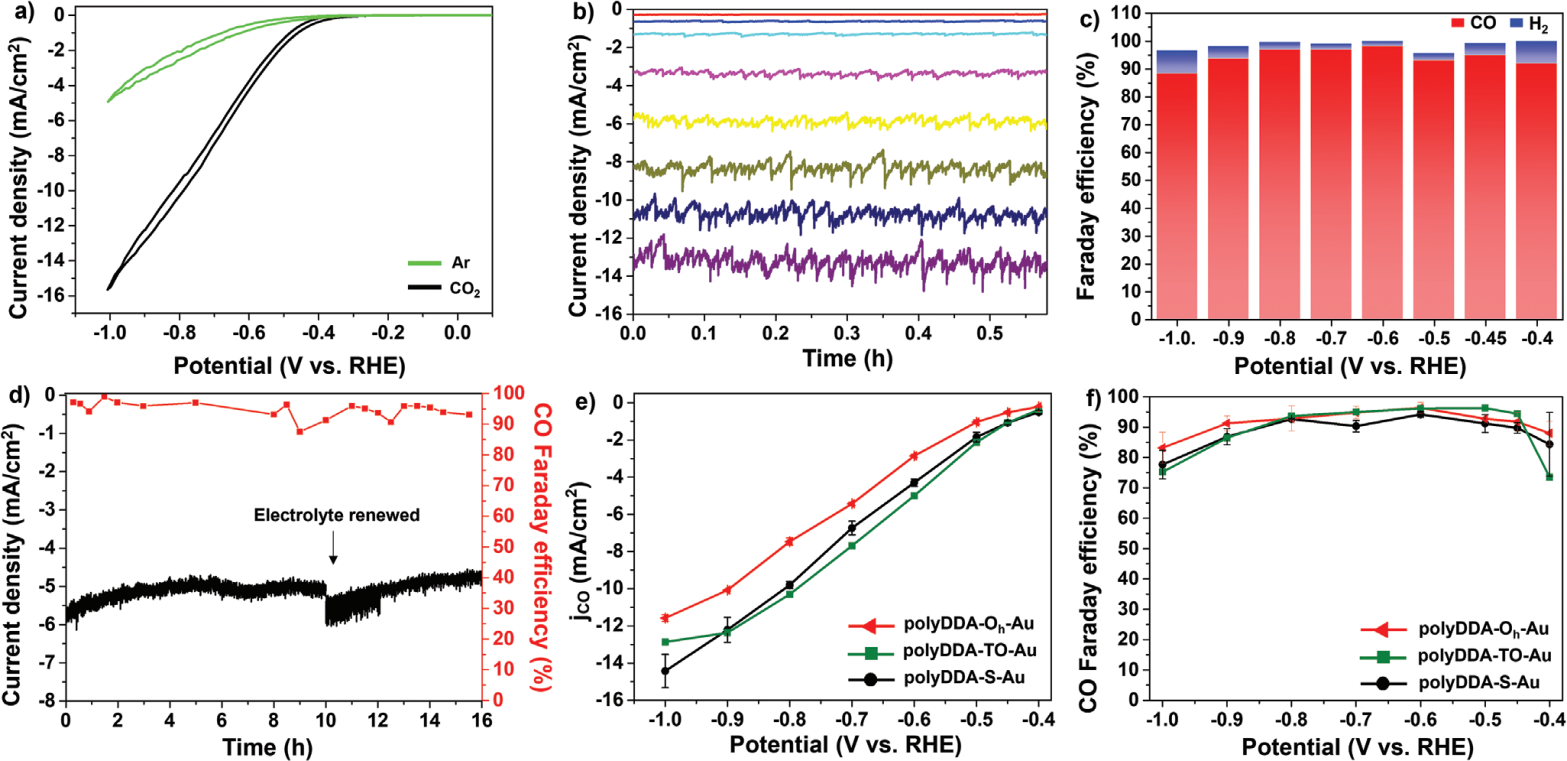
CO_2_RR efficiency of polyDDA‐Au nanoparticles in 0.1 m KHCO_3_. a) CVs in Ar‐ (green) and CO_2_‐saturated (black) electrolyte (0.1 m KHCO_3_, scan rate: 20 mV s^−1^). b) Chronoamperometry curve of polyDDA‐O_h_‐Au and c) Faradaic efficiency (blue: H_2_ and red: CO) depending on applied potentials. d) Chronoamperometric measurement and CO Faradaic efficiency of polyDDA‐O_h_‐Au at an applied potential of −0.7 V versus RHE for 16 h. e) Partial current density of CO and f) CO Faradaic efficiency of polyDDA‐O_h_‐Au (red), polyDDA‐TO‐Au (dark‐green), and polyDDA‐S‐Au (black).

Electrochemical CO_2_RR on the polyDDA‐functionalized Au particles, denoted as polyDDA‐Au, was conducted using an H‐type cell with 0.1 m KHCO_3_ electrolyte and continuous CO_2_ flow, which separates the cathode and anode compartments. The electrocatalytic activity of the as‐prepared 65 nm O_h_‐Au (polyDDA‐O_h_‐Au) on a glassy carbon electrode was preliminarily evaluated using cyclic voltammetry (CV) at a scan rate of 20 mV s^−1^ under Ar‐ and CO_2_‐saturated conditions, respectively (**Figure** **2**a). Under CO_2_‐saturated conditions, the onset potential was shifted in the positive direction compared with that under Ar‐saturated conditions. The generated current was considerably larger under CO_2_‐saturated conditions, indicating that CO_2_ was electrochemically reduced on the polyDDA‐O_h_‐Au surface. The electrocatalytic activity was further investigated using chronoamperometry at constant applied potentials (Figure 2b), and gas products were detected by gas chromatography. CO was a major product and H_2_ was a minor product during the electrocatalytic reaction (Figure 2c); no liquid products were detected by ^1^H NMR. The polyDDA‐O_h_‐Au electrode is capable of reducing CO_2_ to CO with high selectivity over a wide potential range. Typically, the CO Faradaic efficiency was 91.97% even at −0.4 V versus RHE, and reached up to 98.18% at a potential of −0.6 V versus RHE. The CO selectivity of the catalyst remained over 90% at an applied potential of −0.7 V versus RHE for 16 h (Figure 2d, the electrolyte was replaced after 10 h of reaction), indicating that the polyDDA‐O_h_‐Au electrode is highly durable. There are many papers that are efficient in a narrow potential range, but it is not easy to show good efficiency in such a wide potential range.

The structural changes during the CO_2_RR were investigated by acquiring XPS and SEM data after the durability experiment. The surface of the polyDDA‐O_h_‐Au electrode was examined using XPS after the stability test to investigate whether any changes occurred on the Au surface (Figure S6, Supporting Information). XPS analysis showed that the states of N and Cl were almost identical after the CO_2_RR (Figures S3 and S6, Supporting Information), indicating that polyDDA remained bound to the Au surface, even during the long‐term stability test. The SEM image also shows that the octahedral shape was maintained after the electrocatalytic reaction (Figure S7, Supporting Information). Note that the current density of the polyDDA‐O_h_‐Au‐coated carbon paper electrode remained stable for 80 h, and the CO Faradaic efficiency was almost identical over the entire time (Figure S8, Supporting Information). These results confirm the electrocatalytic stability of polyDDA‐O_h_‐Au during the CO_2_RR.

The effect of the size and exposed surface facets of the Au particles for the CO_2_RR was studied.^[^ ^40^ ^]^ To understand the effect of the surface facets of the Au particles, the efficiency and CO selectivity of polyDDA‐TO‐Au and polyDDA‐S‐Au particles were investigated (Figure 2e,f). As mentioned above, polyDDA was still strongly bound to the surfaces of polyDDA‐TO‐Au and polyDDA‐S‐Au during the chemical etching process (Figure S3, Supporting Information). The polyDDA‐TO‐Au and polyDDA‐S‐Au samples showed slightly higher activity for CO generation than polyDDA‐O_h_‐Au (Figure 2e), whereas the CO Faradaic efficiency was almost identical for all samples (Figure 2f). As a result of obtaining the electrochemical surface area (ECSA)^[^ ^41^ ^]^ and comparing the efficiency (Figure S9, Supporting Information), polyDDA‐TO‐Au showed the highest activity, followed by polyDDA‐S‐Au and polyDDA‐O_h_‐Au. The CO selectivity of the polyDDA‐S‐Au catalyst also remained at ≈90% for 17 h at an applied potential of −0.7 V versus RHE (Figure S10, Supporting Information). The shapes of the polyDDA‐TO‐Au and polyDDA‐S‐Au particles were identical after the stability test, and polyDDA was well attached to the Au surface during the CO_2_RR based on XPS analysis (Figures S6 and S7, Supporting Information).

The effect of the particle size (35–150 nm) of polyDDA‐O_h_‐Au on the CO_2_RR was also investigated. The CO generation activity of the small polyDDA‐O_h_‐Au particles (36 nm) was almost identical to that of the 65 nm particles (Figure S11, Supporting Information), and the CO Faradaic efficiency followed a similar trend over the entire potential range for both particle sizes (Figure S11, Supporting Information). Large polyDDA‐O_h_‐Au particles (137 nm) showed slightly decreased activity for CO generation (Figure S11, Supporting Information), but the CO Faradaic efficiency followed a trend identical to that of the small particles. These results indicate that the particle size and shape are not a dominant factor influencing the selectivity in this catalytic system, whereas polyDDA is thought to be the main reason that can affects the CO selectivity on the surface of the Au particles.

**Figure 3:**
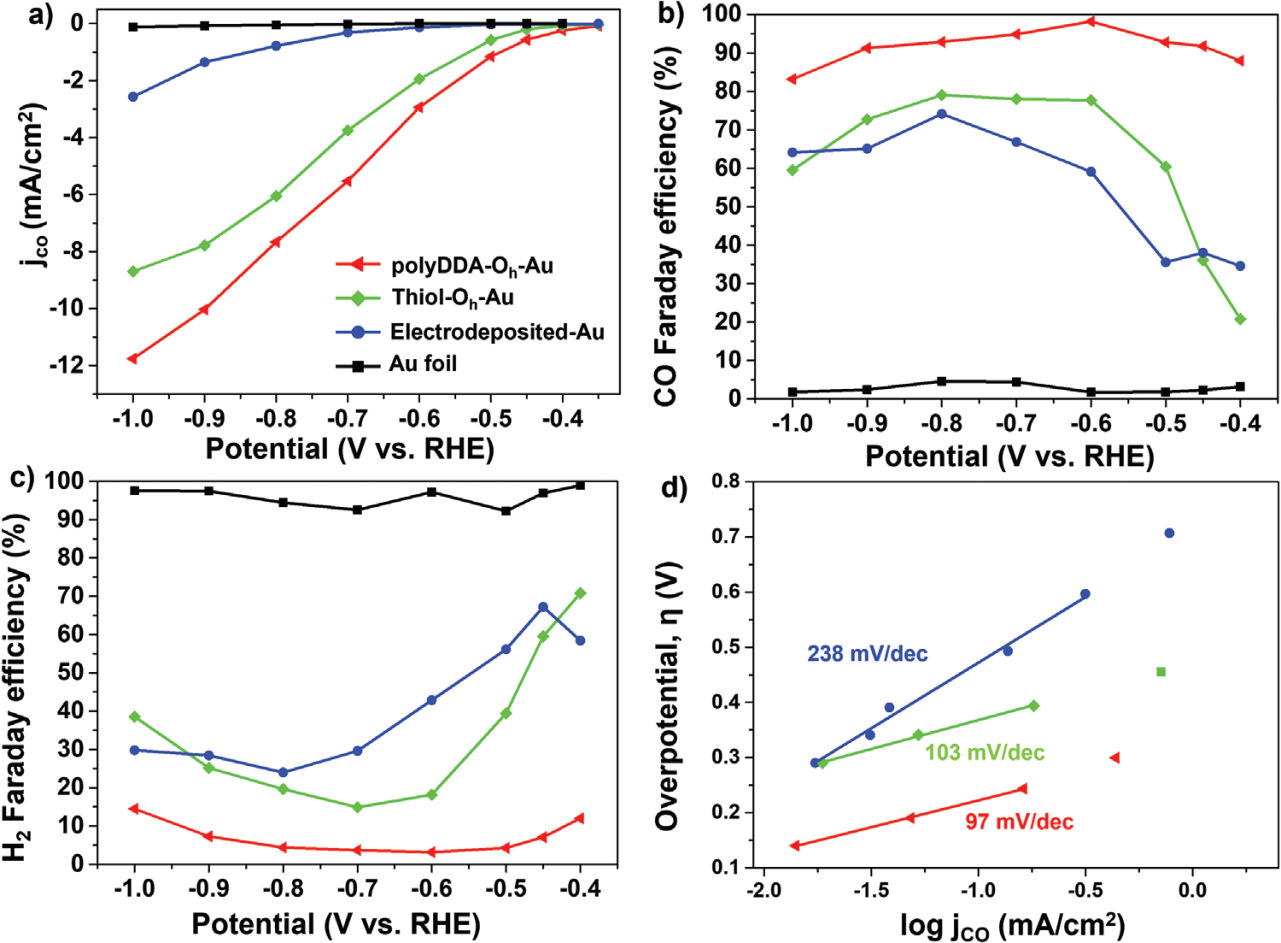
a) Partial current density of CO generated by polyDDA‐O_h_‐Au (red), thiol‐O_h_‐Au (green), electrodeposited‐Au (blue), and Au foil (black) in CO_2_ saturated 0.1 m KHCO_3_. Faradaic efficiency of b) CO and c) H_2_ generated by polyDDA‐O_h_‐Au, thiol‐O_h_‐Au, electrodeposited‐Au, and Au foil electrodes. d) Tafel plots of polyDDA‐O_h_‐Au (red), thiol‐O_h_‐Au (green), and electrodeposited‐Au (blue) electrodes.

To further investigate the origin of the high CO Faradaic efficiency, comparative experiments were conducted by removing polyDDA on the O_h_‐Au surface of the 65 nm particles by annealing the process in an inert condition.^[^ ^30^ ^]^ XPS analysis showed that the peaks of N 1s and Cl 2p diminished, indicating that the ligands were detached to the Au surface (Figure S12, Supporting Information). When polyDDA was removed, the total current density and the partial current density of CO decreased considerably over the whole potential range (Figure S13, Supporting Information). Especially, the CO Faradaic efficiency decreased to 60.43% at −0.4 V versus RHE. Because the shape and size of O_h_‐Au was changed during the annealing process (Figure S12c, Supporting Information), a surfactant exchange was conducted by exchanging polyDDA with the thiol functional group (11‐mercaptoundecanoic acid, thiol‐O_h_‐Au) on the O_h_‐Au surface. The SEM image shows that the particles retained the octahedral shape after the surfactant exchange process (Figure S14a, Supporting Information). XPS analysis showed that the peaks of N 1s and Cl 2p became less intense and a new S 2p peak appeared, indicating that the thiol group was attached to the Au surface (Figure S15, Supporting Information). When polyDDA was exchanged with the thiol group, the total current density decreased slightly (Figure S16, Supporting Information), and the partial current density of CO decreased considerably over the whole potential range (**Figure** **3**a). Typically, the thiol‐O_h_‐Au electrode exhibited CO Faradaic efficiencies of 20.79% and 60.43% at −0.4 and −0.5 V versus RHE, respectively. These values are far below those for the polyDDA‐O_h_‐Au electrode (Figure 3b). The opposite behavior was observed for the HER, and H_2_ was the major product in the low potential region using the thiol‐O_h_‐Au electrode (Figure 3c). The Au electrode without surface functional groups was prepared by the electrodeposition of Au particles (electrodeposited‐Au, Figure S14c, Supporting Information); this electrode led to a trend almost identical to that obtained with the thiol‐O_h_‐Au electrode (Figure 3b,c), even though the absolute current density of the former was far lower (Figure S16, Supporting Information). The Tafel slopes also indicated fast kinetics for polyDDA‐O_h_‐Au (Figure 3d). Note that the CO Faradaic efficiency of the thiol‐functionalized Au sphere (thiol‐S‐Au) electrode was also lower than that of the polyDDA‐S‐Au electrode (Figure S17, Supporting Information). This indicates that the chemically adsorbed polyDDA, dimethylammonium, and chloride ions enhanced the CO selectivity on the surface of the Au particles.

For the high current density, the CO_2_RR was carried out in a gas diffusion electrode (GDE) device using polyDDA‐O_h_‐Au particles. The onset potential was shifted in the positive direction and the generated current was considerably increased under CO_2_‐flow conditions (Figure S18a, Supporting Information). The polyDDA‐O_h_‐Au in GDE improved the mass transfer during the reaction and afforded large operation currents. The CO Faradaic efficiency was 96.22% at −0.3 V and 97.94% even at a potential of −0.8 V versus RHE (Figure S18b, Supporting Information). The CO Faradaic efficiency of the catalyst showed over 96% over a wide potential range. The high CO selectivity over a wide potential range with relatively high current is an exceptional result.

### 2.3 Theoretical Investigation of Electrochemical CO_2_ Reduction Reaction

**Figure 4:**
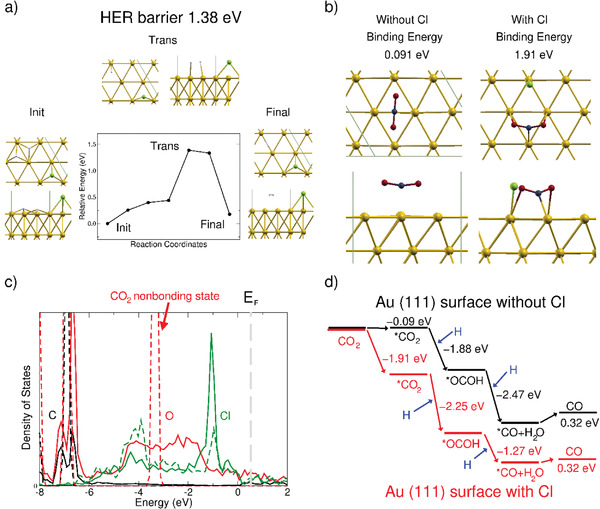
a) NEB calculation results for HER and b) binding energies of CO_2_ on Au (111) with and without Cl. c) PDOS for CO_2_ detached from (dashed‐line) and attached to (solid line) Au (111) with surface Cl and d) energy diagram during CO_2_ reduction reaction on Au (111) with and without surface Cl. Top and side views of each structure are shown in upper and lower positions. Blue, black, yellow, red, and green balls represent H, C, Au, O, and Cl atoms, respectively. Fermi level is indicated by dashed gray line in (c). * represents adsorbed molecules.

DFT calculations were conducted to further understand the effect of the polyDDA group on the Au surface. Because Cl atoms on the Au surface drastically enhanced the CO_2_RR activity, the role of Cl atoms on the Au (111) surface was studied to elucidate the reaction mechanism. Given the high CO_2_RR activity on the Cl‐functionalized Au (111) surface, the energy barrier for the CO_2_RR should be reduced and should be lower than that for the HER. For comparison, the HER barrier was determined using nudged elastic band (NEB) calculations (**Figure** **4**a).

The formation of H_2_ molecules and their release from the surface occurred simultaneously with an HER barrier of 1.38 eV with a surface Cl atom, which is much higher than without a Cl atom (Figure S19, Supporting Information). The binding energies of CO_2_ molecules on the Au (111) surfaces with and without Cl atoms were also calculated (Figure 4b). The binding energies and structural deformations of CO_2_ molecules on the Au (111) surface with surface Cl atoms (hereafter Au111‐Cl) are listed in **Table** **1**. The binding energy was 1.91 eV and the CO_2_ height was 1.768 Å, where the latter value is considerably smaller than that of bare Au (111), revealing strong interaction between Au111‐Cl and the CO_2_ molecule. The large CO_2_ binding energy of 1.91 eV also indicates that the initial CO_2_ adsorption is fast and CO_2_ is strongly adsorbed on Au111‐Cl.

**Table 1:** Binding energies and geometric parameters for CO_2_ adsorption on Au (111) surface with and without Cl. CO_2_ vertical height is measured between carbon atom and topmost Au atoms

Au (111)	CO_2_ height [Å]	2.11
	Binding energy [eV]	0.091
	O—C—O angle [°]	164
Au (111)‐Cl	CO_2_ height [Å]	1.768
	Binding energy [eV]	1.91
	O—C—O angle [°]	140

To understand the large binding energy of CO_2_ on Au111‐Cl, the partial density of states (PDOS) was plotted, as shown in Figure 4c. The dashed and solid lines represent the PDOS of the detached and attached CO_2_ molecules on Au111‐Cl. The PDOS of C, O, and Cl are shown in black, red, and green colors, respectively. The Au PDOS plots (not shown in Figure 4c) were dominant and much larger in the energy range. The nonbonding states of the non‐adsorbed CO_2_ molecule were located at −3.3 eV (Figure 4c).

Upon adsorption, the nonbonding states were hybridized with Cl‐induced Au states; the sharp peak around −3.3 eV overlapped with the peaks of the Cl states, as indicated by the red solid lines. Covalent bonding between the nonbonding states of the CO_2_ molecule and the Cl‐induced Au state led to the large binding energy of 1.91 eV. Notably, there was no direct bonding between Cl and the adsorbed CO_2_ molecule during the reaction (Figure 4b). Subsequent calculations for hydrogenation of CO_2_ adsorbed on Au111‐Cl were conducted to evaluate the fast CO_2_RR. Hydrogen (H^+^ + e^−^) atoms were inserted in the form of H_3_O^+^ cations. In the presence of the Au substrate, Bader charge analysis revealed that H_3_O^+^ is cationic due to electron transfer to Au. Because the Au substrate is metallic, it is not expected to have a significant effect on the hydrogenation reaction involving electron transfer to Au. The first and second hydrogenation processes are shown in Figures S20 and S21, Supporting Information, respectively. The H_3_O^+^ molecule is located near the surface‐attached CO_2_ and COOH molecules on Au111‐Cl. The structural optimization of the *COOH and *CO + H_2_O molecules after the first and second hydrogenations indicated that the energy was lowered by −2.25 and −1.27 eV, respectively, as shown in Figure 4d (red color). Here, * represents surface adsorption. All the reaction steps from CO_2_ adsorption to the second hydrogenation are energetically downhill reactions on the Au111‐Cl surface. The last step for CO production is the release of the CO molecule from the surface, with an activation energy of 0.32 eV, which is comparable to the values from previous experiments and calculations.^[^ ^42^, ^43^ ^]^ The value is less than one fourth of the HER barrier of 1.38 eV, which results in high CO selectivity and reactivity on the polyDDA‐Au surface. In contrast, the initial CO_2_ adsorption was minimal due to the weak binding energy (0.091 eV) on the Au surface without Cl; this in turn limits the CO_2_RR. The hydrogenation processes to surface CO_2_ are similar (Figures S22 and S23, Supporting Information). An energy diagram for the case without Cl atoms is shown in Figure 4d (black color). Despite a general similarity of hydrogenation processes, CO_2_RR with pure Au (111) surface is unlikely since the CO_2_ binding energy is so weak and therefore a negligible number of CO_2_ molecules will react with H ions. And thus the low‐barrier of HER will be preferred, which matches well with the experimental results (Figure 3 and Figure S13, Supporting Information).

The Cl atoms on the Au surface enhance the CO_2_RR selectivity and activity compared with those of bare Au, but the Cl‐modified Au shows poor stability due to the desorption of Cl^−^ ions during continuous CO_2_RR.^[^ ^29^ ^]^ In this study, however, the polyDDA‐Au particles showed drastically enhanced stability. This indicates that the chemically adsorbed polyDDA, that is, the dimethylammonium moiety, affected the stability of Cl adsorbed on the Au surface. To investigate the possibility of Cl release, DFT calculations were conducted for the vertical approach of K atoms (from the electrolyte) toward the Cl atoms with and without surrounding 8H_2_O molecules, as shown in Figure S24, Supporting Information. In any case, Cl atoms were extracted from the surface as KCl, proving the instability of Cl during the reaction. In this sense, the polyDDA group plays two positive roles: supplying and stabilizing the Cl atoms on the Au surface. The polyDDA group supplies Cl atoms, which changes the surface properties Au (111) to induce strong CO_2_ adsorption. DFT calculations were conducted to investigate the transfer of Cl from polyDDA to the Au (111) surface. polyDDA is composed of Cl anions and organic polymer cations, and the organic polymer component interacts slightly with the Au surface. Geometrically, the only difference is that the Cl anion is attached to polyDDA or on the Au (111) surface away from polyDDA. When the Cl anion is attached to the Au (111) surface, the total energy is lowered by 0.5 eV based on DFT calculations. The calculation results support the role of polyDDA in supplying Cl on the Au surface. In addition, polyDDA increases the stability of Cl adsorbed on the Au surface owing to the positive charge of the dimethylammonium group (Figure S25, Supporting Information). The dimethylammonium moiety as a surfactant undergoes consecutive coulombic interactions with adsorbed Cl, preventing its release from the Au surface, thus enhancing the CO_2_RR activity and stability compared with those of bare Au.

### 2.4 The polyDDA Functionalization to Other Metal Catalysts for Electrochemical CO_2_ Reduction

**Figure 5:**
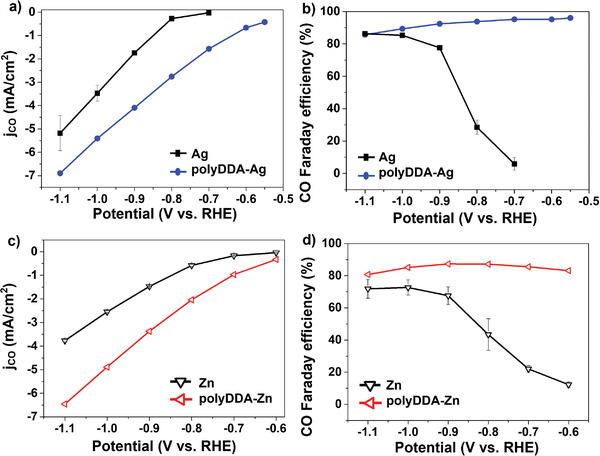
a) Partial current density of CO and b) CO Faradaic efficiency of Ag and polyDDA‐Ag in CO_2_‐saturated 0.1 m KHCO_3_. c) Partial current density of CO and d) CO Faradaic efficiency of Zn and polyDDA‐Zn electrodes in CO_2_‐saturated 0.1 m KHCO_3_.

To expand the application range of polyDDA functionalization to other metal electrodes, polyDDA‐functionalized Ag (polyDDA‐Ag) and Zn (polyDDA‐Zn) electrodes were prepared via a simple adsorption process using polyDDA solution. The onset potential of the polyDDA‐Ag electrode was shifted in the positive direction, and the partial current density of CO increased significantly over the entire potential range (**Figure** **5**a). The polyDDA‐Ag electrode exhibited a CO Faradaic efficiency of 95% at −0.8 V versus RHE, which is much higher than that of the untreated Ag electrode (22% at −0.8 V versus RHE) (Figure 5b). The CO selectivity of polyDDA‐Ag remained over 95% at an applied potential of −0.8 V versus RHE for 5 h (Figure S26a, Supporting Information), indicating that the polyDDA‐Ag electrode has high durability. The partial current density of CO and durability of the polyDDA‐Zn electrode were also highly improved (Figure 5c and Figure S26b, Supporting Information) and the CO Faradaic efficiency was enhanced over the entire potential range (Figure 5d), confirming that the chemically adsorbed polyDDA positively affected the CO selectivity on diverse metal surfaces.

## 3 Conclusion

The polyDDA‐functionalized Au particles were prepared for selective electrochemical CO_2_ reduction. Single‐crystalline Au particles, O_h_‐Au, TO‐Au, and S‐Au, were synthesized by a consecutive process of growth and chemical etching in the presence of a polyDDA surfactant. The polyDDA‐Au electrode showed high catalytic activity and durability for CO production. The Faradaic efficiency of this electrode exceeded 90% for CO production in the range between −0.4 and −1.0 V (versus RHE), reaching up to 98% at −0.6 V in the H‐type cell with 0.1 m KHCO_3_ electrolyte. In addition, the polyDDA‐Au particles in GDE under CO_2_‐flow conditions afforded large operation currents, and the CO Faradaic efficiency of the catalyst showed over 96% over a wide potential range. The high CO selectivity in a wide potential range with a relatively high current is an exceptional result. The influence of polyDDA coated on Au particles and the origin of the enhanced activity and stability were fully investigated using theoretical studies. Chemically adsorbed dimethylammonium and chloride ions were bound to the Au surface, which consecutively affected the initial adsorption of CO_2_ and the stability of the *CO_2_, *COOH, and *CO intermediates during continuous CO_2_RR. The dimethylammonium moiety as a surfactant undergoes consecutive coulombic interactions with adsorbed Cl, preventing its release from the Au surface, thus enhancing the CO_2_RR activity and stability compared with those of bare Au. Interestingly, polyDDA functionalization can be widely applied to other metal catalysts such as Ag and Zn, which also exhibit improved CO selectivity and stability compared with the untreated counterparts. This approach affords broad applicability for utilizing metal catalysts for efficient CO_2_ reduction reactions.

## 4 Experimental Section

### Selective Synthesis of polyDDA‐Functionalized Au Particles

PolyDDA‐functionalized octahedral Au nanoparticles were obtained by a solvothermal reaction, by following a reported method with some modifications. PolyDDA solution (0.4 g) and aqueous phosphoric acid (0.8 mL) were dissolved in 22 g of ethylene glycol. The solution was stirred for 30 min after adding 20 µL of 0.5 m HAuCl_4_·4H_2_O. The prepared solution was heated at 200 °C for 30 min.

### Etching Octahedral Particles to Form Truncated Octahedral and Spherical Au Particles

Monodispersed truncated octahedral Au and spherical Au particles were prepared by a consecutive etching process using a suspension of octahedral Au particles. To prepare the truncated octahedral Au, 0.05 m HAuCl_4_·4H_2_O (19 µL) aqueous solution was introduced into the suspension of octahedral Au. The mixture was then stirred for 24 h to obtain truncated octahedral Au particles. For spherical Au, 0.5 m HAuCl_4_·4H_2_O (12.5 µL) was introduced into the suspension of octahedral Au particles, and the solution was stirred for 24 h to obtain spherical Au particles.

### Ligand Exchange from polyDDA to 11‐Mercaptoundecanoic Acid

The polyDDA‐functionalized Au particles were added to 5 mL 0.2 m 11‐mercaptoundecanoic acid solution. The solution was stirred for 17 h at 70 °C to obtain the thiol‐functionalized Au nanoparticles.

### Electrodeposition of Au Nanoparticles

Electrodeposition was performed using chronoamperometry at an applied potential of −0.2 V (versus RHE) for 1000 s in 2.5 mm HAuCl_4_·4H_2_O electrolyte solution on a glassy carbon plate.

### PolyDDA‐Functionalized Zn and Ag Electrode

Ag or Zn nanoparticles were mixed with Nafion solution (10 mg mL^−1^) to make the catalyst ink, and 100 µL of the catalyst ink was cast on the carbon paper. The Ag (or Zn)‐loaded carbon paper was dipped in 3.5 wt% polyDDA solution for 30 min and then washed in DI water to remove any residual polyDDA.

### Statistical Analysis

The average size and standard deviation of Au nanoparticles in Figure 1 and Figure S1, Supporting Information, were determined by measuring at least 100 particles. In Figures 2 and 5, and Figures S9, S11, and S17, Supporting Information, the error bars represent the standard deviation based on at least three measurements. All plotting and analysis were conducted with Origin 9.0.

## Conflict of Interest

The authors declare no conflict of interest.

## Supporting information

Supporting Information

## Data Availability

Research data are not shared.
