# Supplementary material for: Unraveling the Simultaneous Enhancement of Selectivity and Durability on Single‐Crystalline Gold Particles for Electrochemical CO2 Reduction
Source: Adv Sci (Weinh). 2022 May 2;9(20):2201491. doi: 10.1002/advs.202201491 (PMC9284124; doi:10.1002/advs.202201491)
Supplement: Supplementary file 1 — Supporting Information [file ADVS-9-2201491-s001.pdf]

ADVANCED SCIENCE
Open Access

Supporting Information

for *Adv. Sci.*, DOI 10.1002/advs.202201491

Unraveling the Simultaneous Enhancement of Selectivity and Durability on Single-Crystalline Gold Particles for Electrochemical CO_2 Reduction

Yun Ji Lim, Dongho Seo, Syed Asad Abbas, Haeun Jung, Ahyeon Ma, Kug-Seung Lee, Gaehang Lee, Hosik Lee* and Ki Min Nam**

Supporting Information

Unraveling the Simultaneous Enhancement of Selectivity and Durability on Single-Crystalline Gold Particles for Electrochemical CO_2 Reduction

Yun Ji Lim, Dongho Seo, Syed Asad Abbas, Haeun Jung, Ahyeon Ma, Kug-Seung Lee, Gaehang Lee, Hosik Lee*, and Ki Min Nam**

Y. J. Lim, D. Seo, S. A. Abbas, H. Jung, A. Ma, Prof. K. M. Nam*
Department of Chemistry and Chemistry Institute for Functional Materials, Pusan National University, Geumjeong-gu, Busan 46241, Republic of Korea
E-mail: namkimin.chem@gmail.com

Dr. K. Lee
8C Nano Probe XAFS Beamline, Pohang Accelerator Laboratory, Pohang 37673, Republic of Korea

Dr. G. Lee*
Korea Basic Science Institute (KBSI), Daejeon 34133, Republic of Korea
E-mail: ghlee@kbsi.re.kr

Dr. H. Lee*
Department of Energy Engineering, School of Energy and Chemical Engineering, Ulsan National Institute of Science and Technology (UNIST), Ulsan 44919, Republic of Korea
E-mail: hosiklee@gmail.com

Detailed methods

1. Materials

$HAuCl_4 \cdot 4H_2O$ (99.33%, Kojima Chemicals) was used as the metal precursor salt. Ethylene glycol (≥99%, Daejung), poly(diallydimethylammonium chloride) solution (M_w = 400k–500k, 20 wt% in H_2O, polyDDA, Sigma-Aldrich), 11-mercaptoundecanoic acid (95%, Sigma-Aldrich), phosphoric acid (Daejung), zinc (nanopowder, 40−60 nm, ≥99% trace metal base), and silver (nanopowder, <150 nm particle size, trace metal base) were used as received. Deionized water was used as the solvent in all electrochemical experiments. A glassy carbon plate (DASOMRMS), carbon paper (MGL190, AvCarb), and Au foil (99.99%, DASOMRMS) were used as substrates.

2. Characterization

The samples were characterized by high-resolution scanning electron microscopy (HR-SEM, ZEISS SUPRA 25) and field-emission transmission electron microscopy (FE-TEM, TALOS F200X), with acquisition of the selected area electron diffraction patterns. The XRD patterns were acquired using Cu-K_α radiation at 40 kV and 300 mA (Rigaku, D/MAX-2500). XPS measurements were performed using a K-alpha spectrometer with an Al-Kα X-ray source and a pass energy level of 40 eV.

3. Electrochemical measurements

The electrochemical properties were studied by CV and chronoamperometry. CV was performed at a scan rate of 20 mV/s (CHI604 Austin, TX, Ivium V89128 Vertex.One). The Au catalysts (5.5 mg/cm^2) loaded on a glassy carbon plate (10 mm × 20 mm × 1 mm (Th)) or Ag and Zn catalysts (1 mg/cm^2) loaded on carbon paper served as the working electrode; coiled Pt was used as the counter electrode, and a saturated calomel electrode was used as the reference electrode. Electrochemical measurements were performed in 0.1 M $KHCO_3$ (99.5–100.5%; Daejung) aqueous solution in a three-electrode H-type cell separated by a Nafion 115 membrane. The flow rate of CO_2 was maintained at 10 cm^3/min, and the electrolyte was continuously stirred at 500 rpm during the chronoamperometry measurements. During the electrochemical reduction of CO_2, gaseous products were detected using a gas chromatograph (6500GC System; YL Instrument Co.) equipped with a Carboxen-1000 column, thermal

conductivity detector, flame ionization detector, and a methanizer with Ar (99.999%) as the carrier gas.

4. Computational details

All calculations were carried out in the framework of spin-polarized DFT using the Vienna ab initio simulation package with the projector-augmented wave method [1,2]; the generalized gradient approximation of Perdew–Burke–Ernzerhof (PBE) was used to consider the exchange correlation [3]. The cut-off energy for the plane-wave basis set was 500 eV, and the ionic positions of all structures were relaxed until the force converged to below 0.01 eV·Å^{-1}. A k-point mesh of the Γ-centered 3 × 3 × 1 set was adopted. The vacuum between slabs along the *z*-direction was set to a minimum of 14 Å to minimize interactions between the slabs and reduce the complexity of the calculation. The NEB method was used to calculate the migration barriers. A unit cell with 3 × 3 × 1 Au (111) surfaces and seven Au layers was adopted for all calculations. For some calculations, van der Waals interactions were considered, but no significant difference was found.

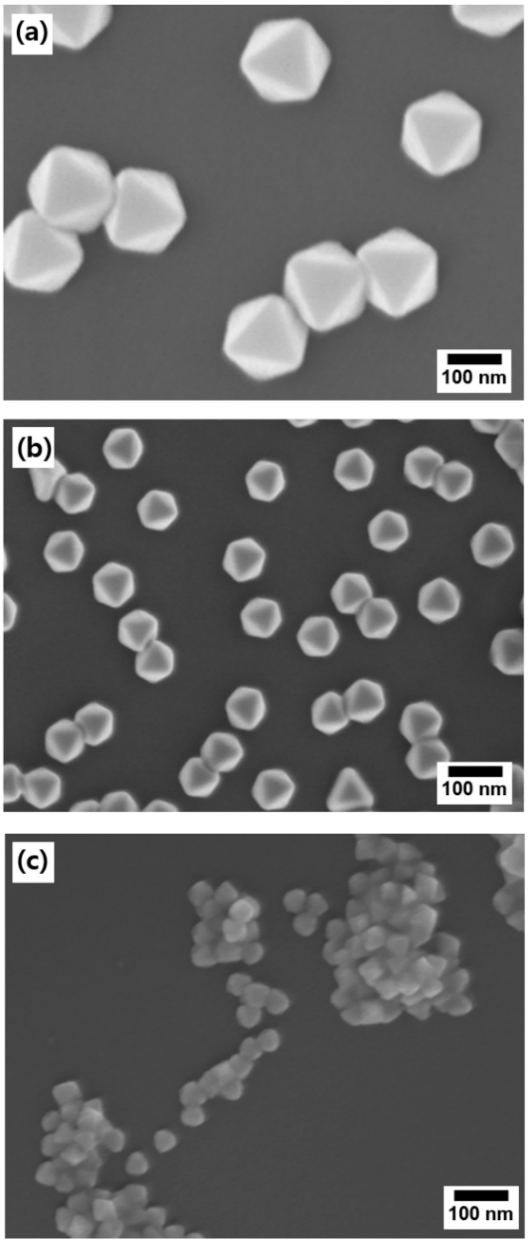

Figure S1. SEM images of O_h-Au particles of (a) 137.6 ± 4.7, (b) 65.4 ± 2.2, and (c) 36.1 ± 0.4 nm.

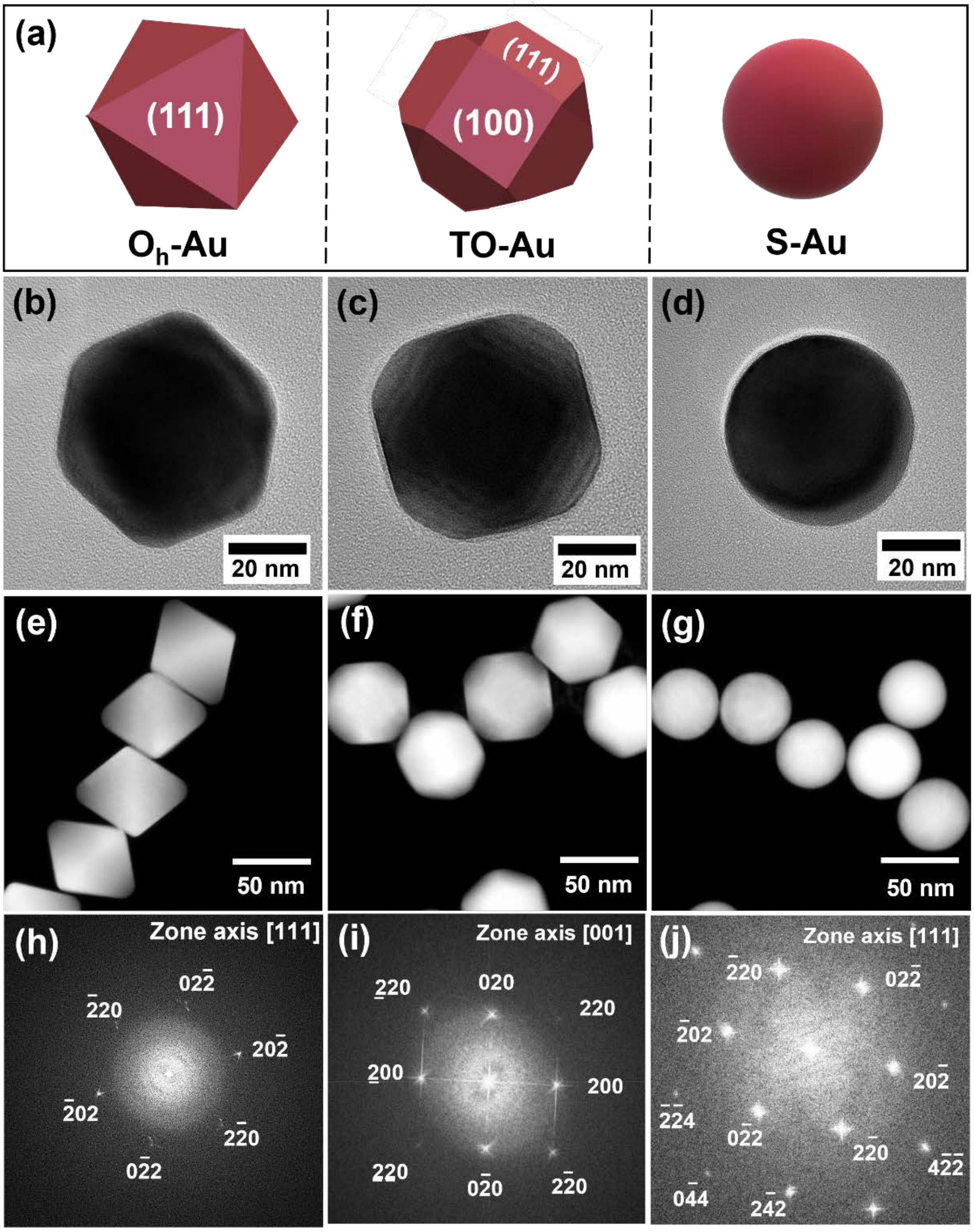

Figure S2. (a) Schematic image, (b-d) HR-TEM images, (e-g) HADDF images and (h-j) FFT images of O_h-Au, TO-Au and S-Au.

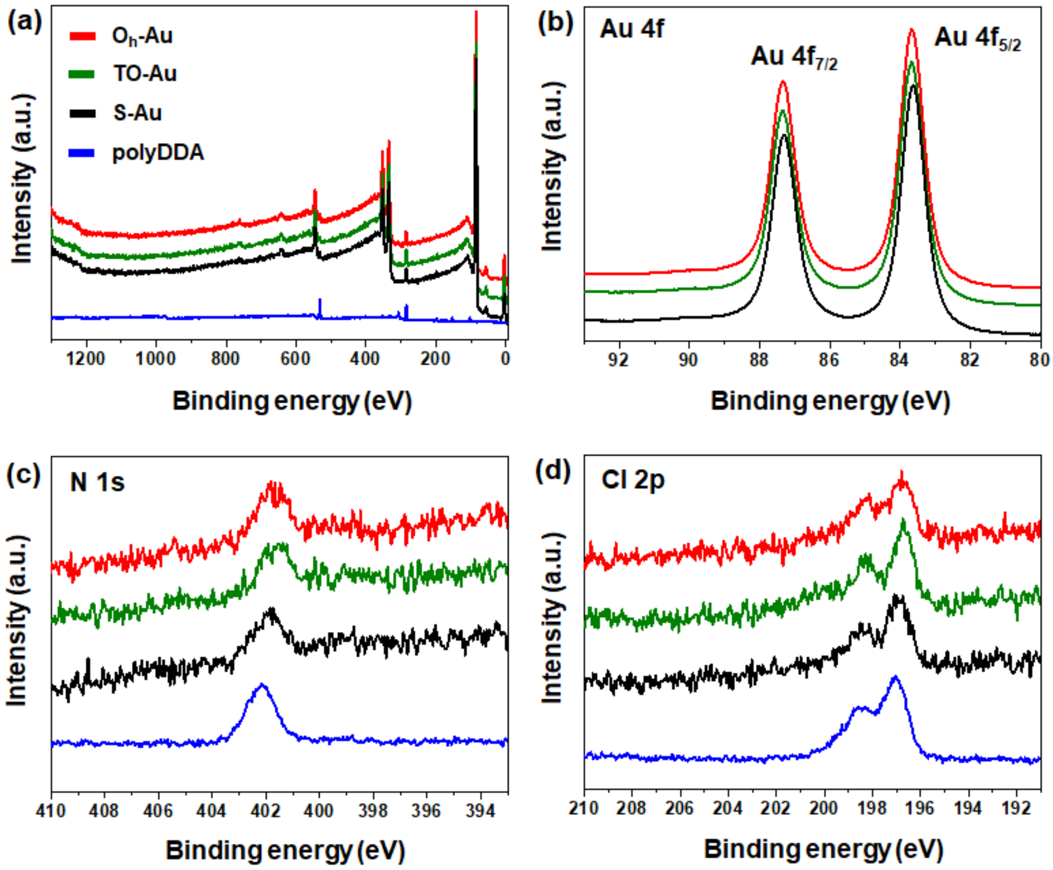

Figure S3. (a) XPS survey spectra, and high-resolution XPS spectra of (b) Au 4f, (c) N 1s, (d) Cl 2p of O_h-Au, TO-Au, S-Au, and polyDDA.

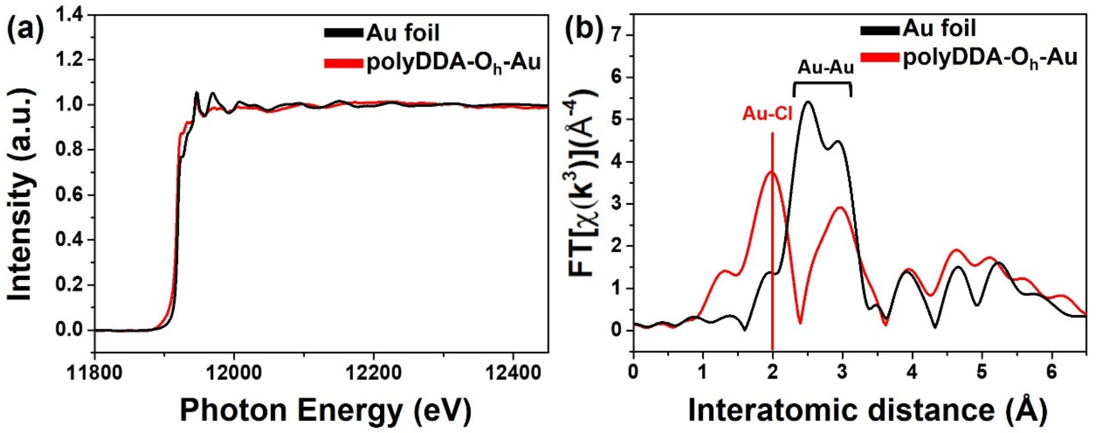

Figure S4. (a) XANES spectra at the Au L-edge, and (b) Radial distribution function obtained by Au L-edge EXAFS spectra of polyDDA-O_h-Au and Au foil.

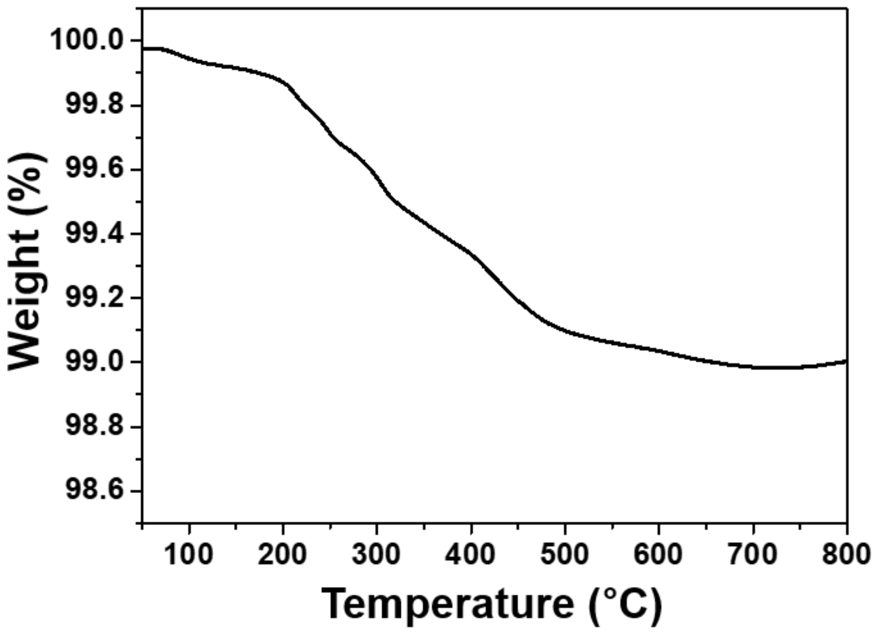

Figure S5. Thermogravimetric analysis (TGA) data of polyDDA-O_h-Au from 50 °C to 800 °C in air.

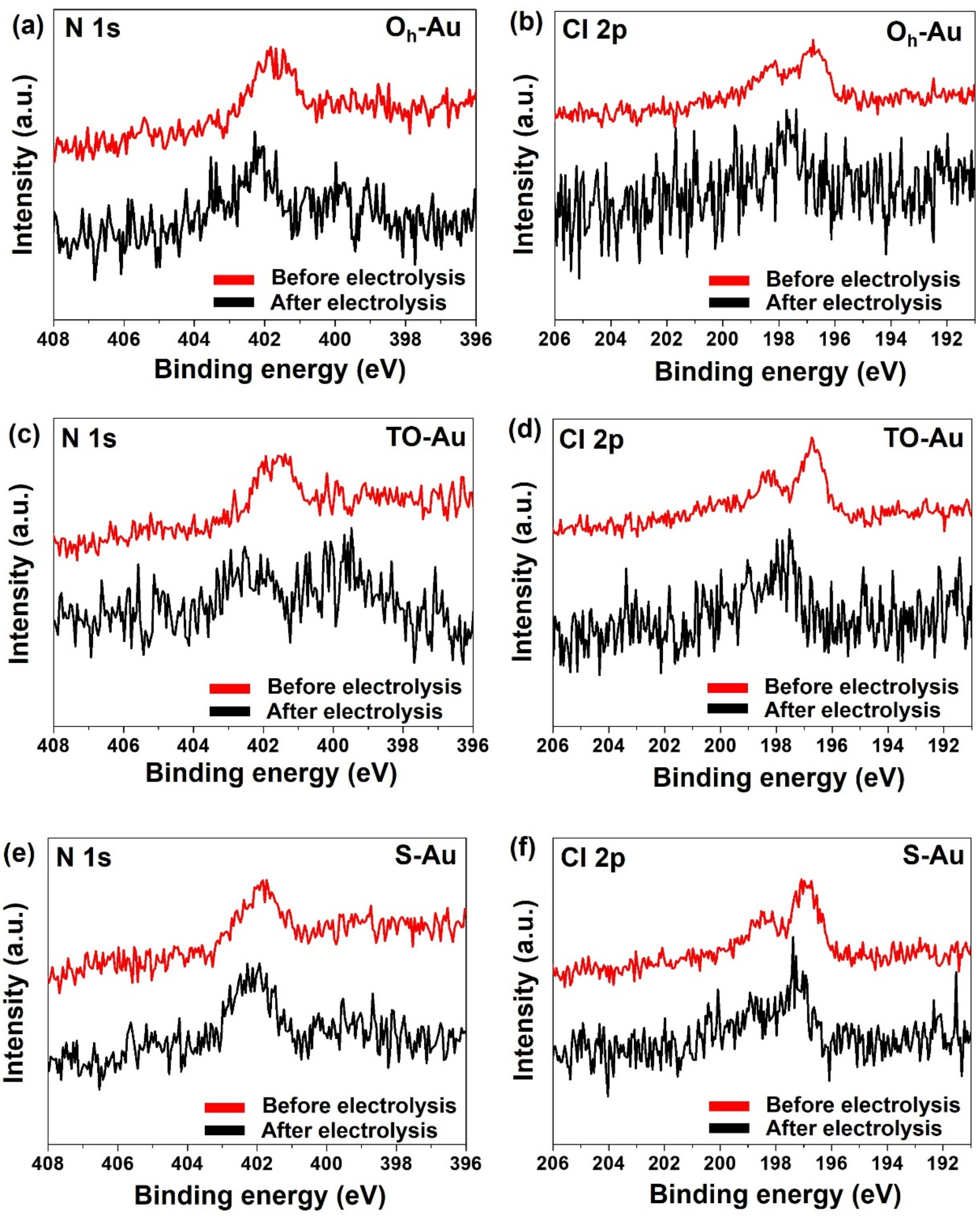

Figure S6. High-resolution XPS spectra of (a-b) polyDDA-O_h-Au, (c-d) polyDDA-TO-Au, and (e-f) polyDDA-S-Au for N 1s and Cl 2p before and after CO_2RR.

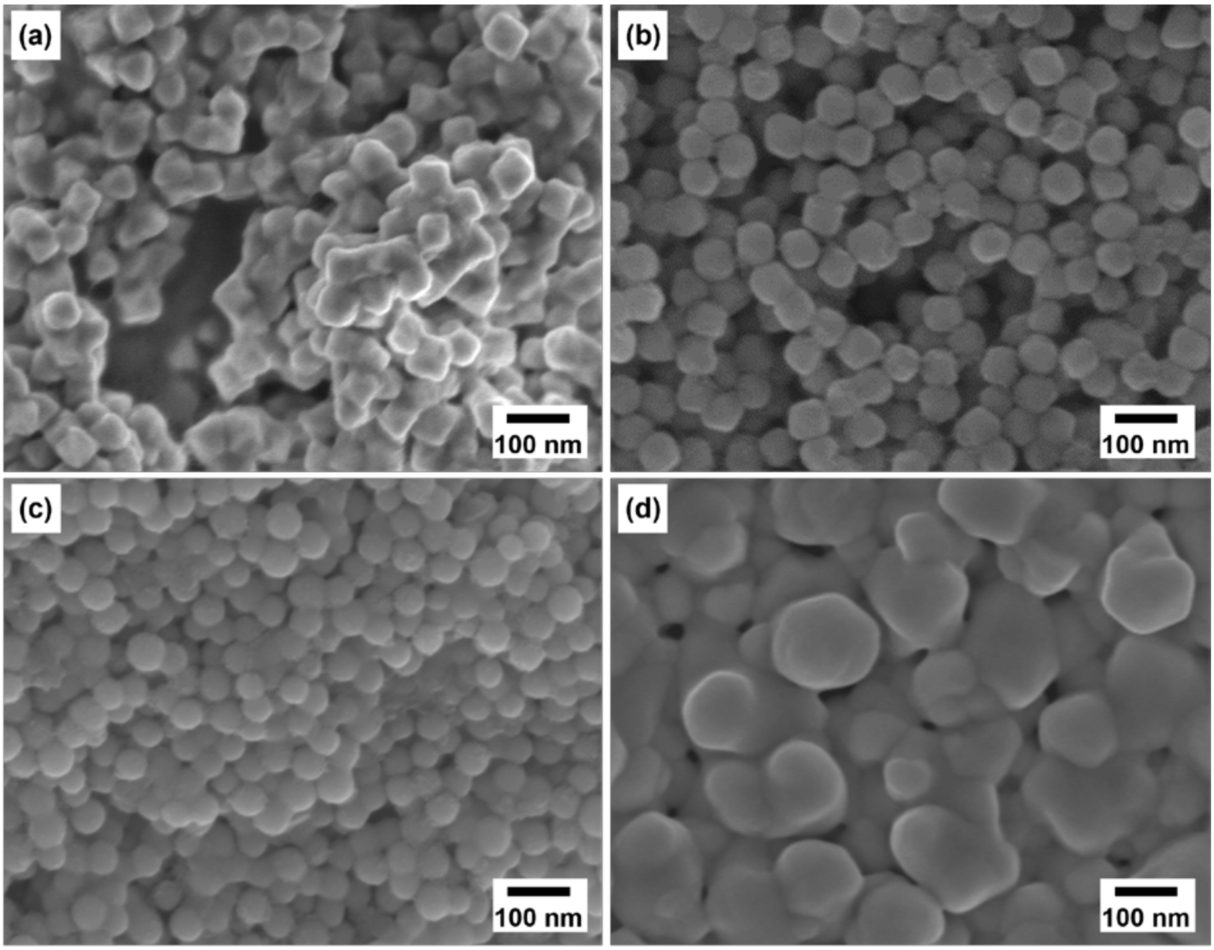

Figure S7. SEM images of (a) polyDDA-O_h-Au, (b) polyDDA-TO-Au, (c) polyDDA-S-Au, and (d) Electrodeposited-Au after CO_2RR.

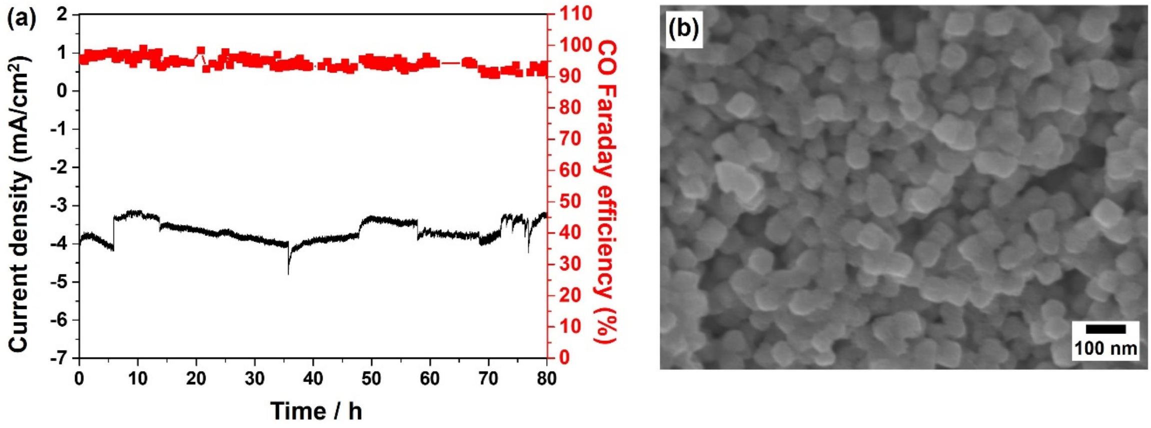

Figure S8. (a) Chronoamperometric measurement and CO Faraday efficiency of polyDDA-O_h-Au on carbon paper electrode at an applied potential of -0.6 V (vs. RHE) for 80 h. (b) SEM image of polyDDA-O_h-Au after CO_2RR on carbon paper electrode.

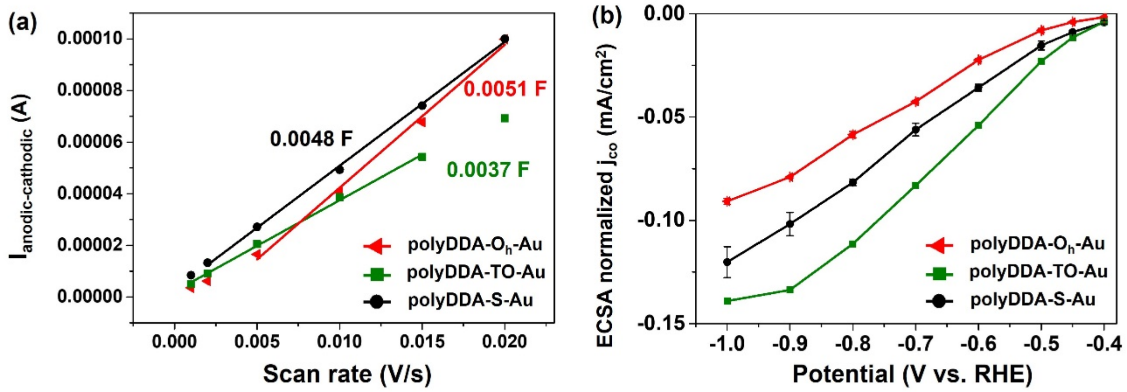

Figure S9. (a) Double layer capacitance of polyDDA-TO-Au, polyDDA-S-Au and polyDDA-Oh-Au and (b) catalytic activity based on ECSA.

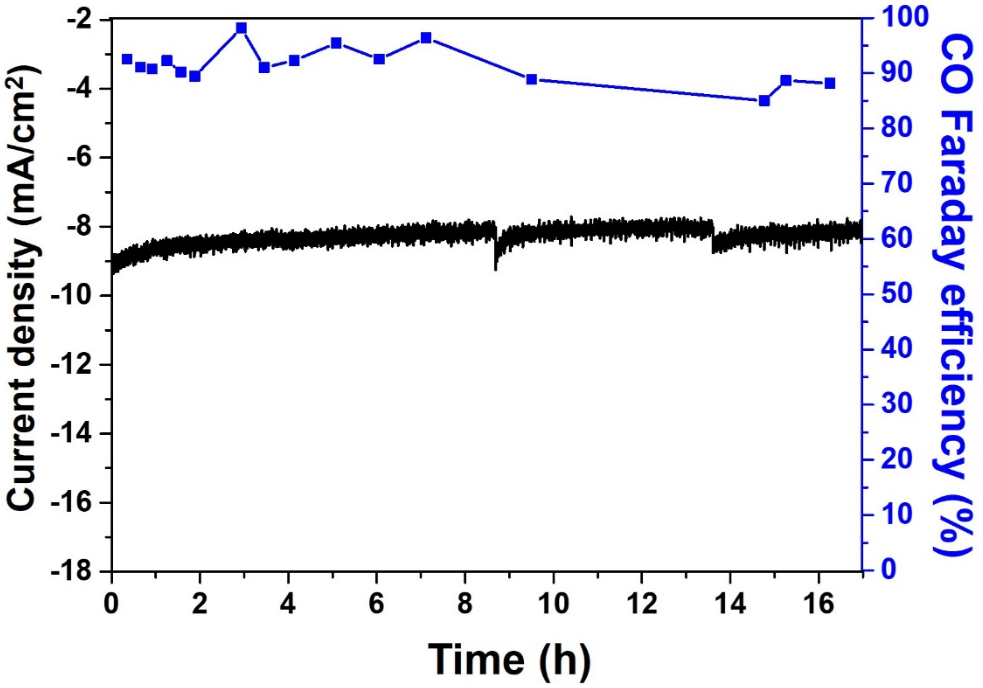

Figure S10. Chronoamperometric measurement and CO Faraday efficiency of polyDDA-S-Au on glassy carbon at an applied potential of -0.7 V (vs. RHE) for 17 h.

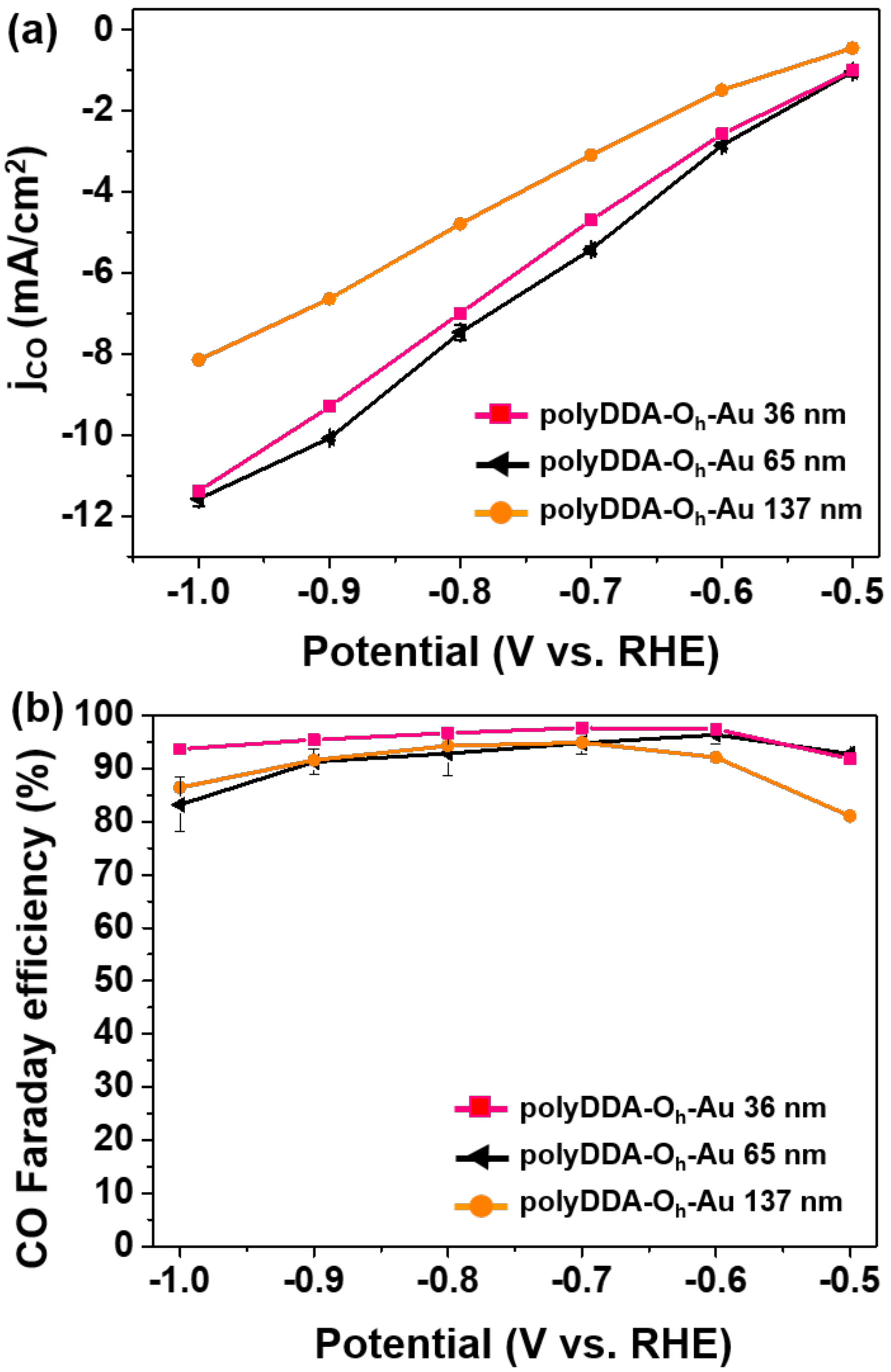

Figure S11. (a) Partial current density of CO, and (b) CO Faraday efficiency of 36 (red), 65 (black), and 137 nm (orange) of polyDDA-O_h-Au.

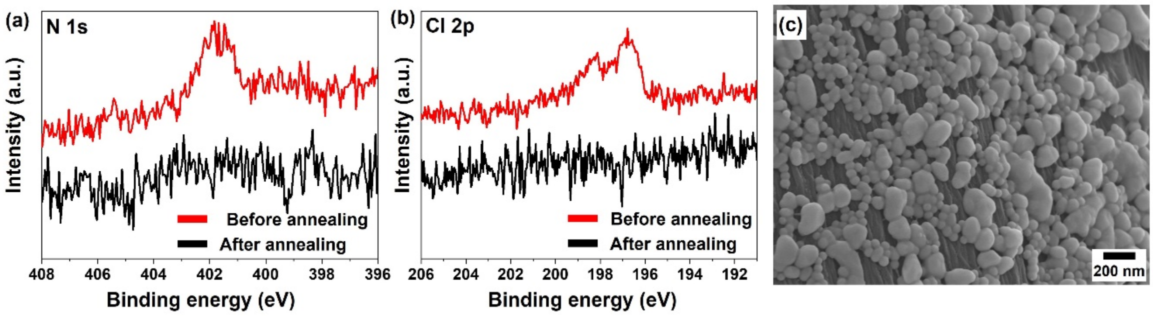

Figure S12. High-resolution XPS spectra of (a) N 1s, and (b) Cl 2p for O_h-Au before and after annealing process. (c) SEM images of O_h-Au after annealing process on carbon paper.

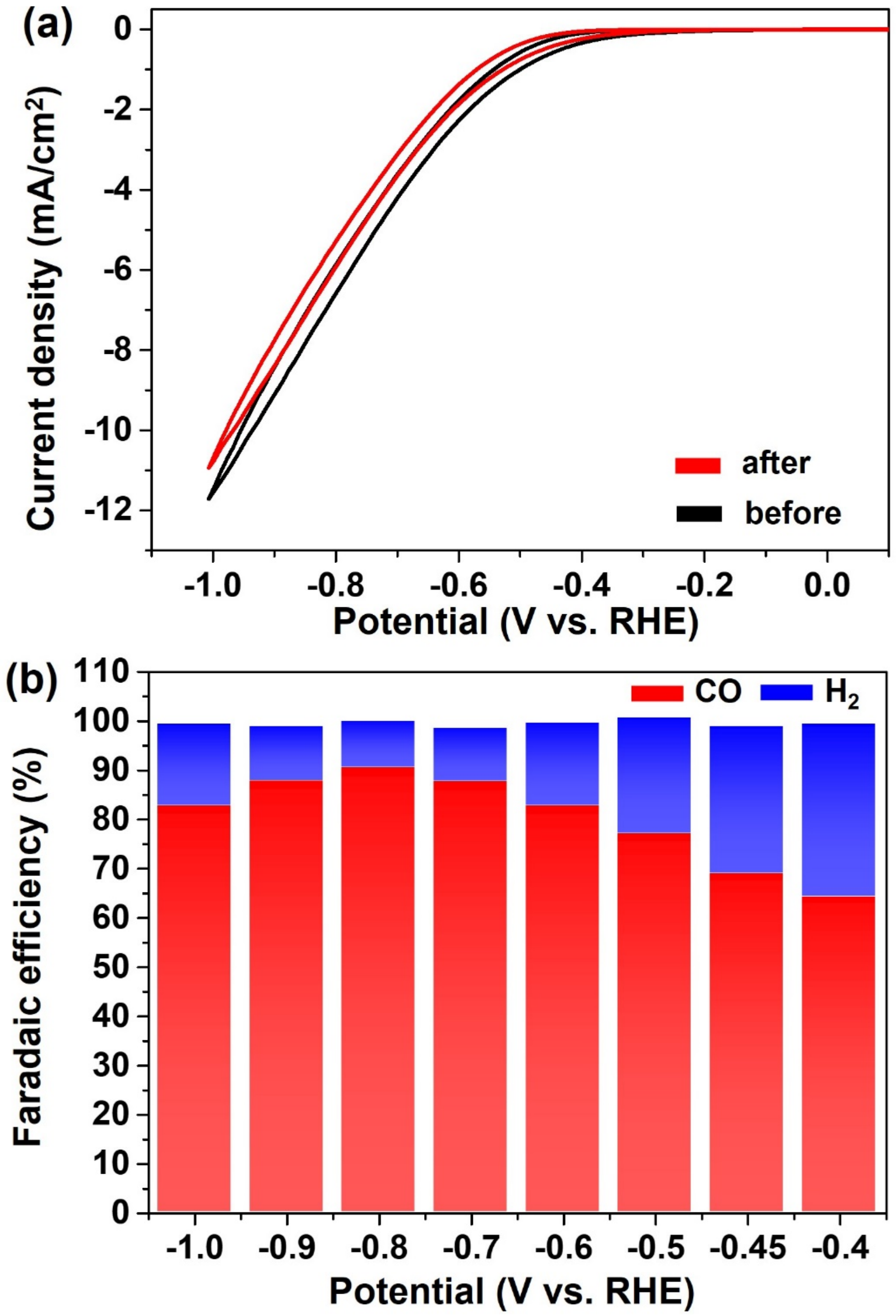

Figure S13. (a) CVs in CO_2-saturated electrolyte (0.1 M $KHCO_3$, scan rate: 20 mV/s), and (b) Faradaic efficiency (blue: H_2 and red: CO_2) depending on applied potentials after annealing process on carbon paper.

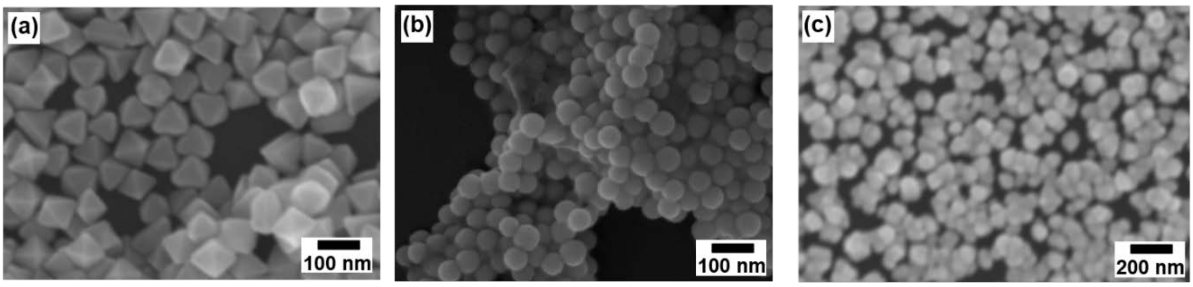

Figure S14. SEM images of (a) Thiol-O_h-Au, (b) Thiol-S-Au and (c) Electrodeposited-Au.

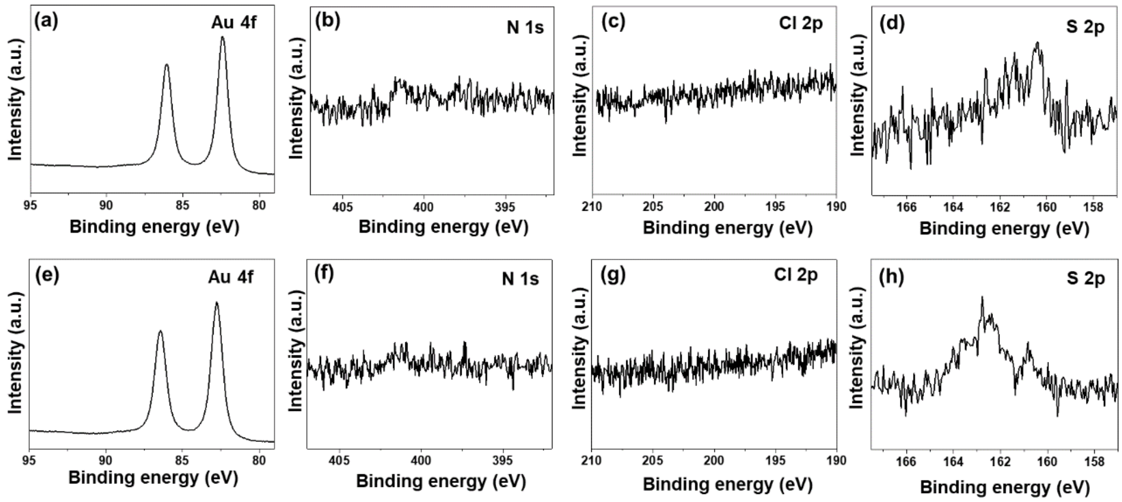

Figure S15. High-resolution XPS spectra of Au 4f, N 1s, Cl 2p and S 2p for (a-d) Thiol-O_h-Au, and (e-h) Thiol-S-Au.

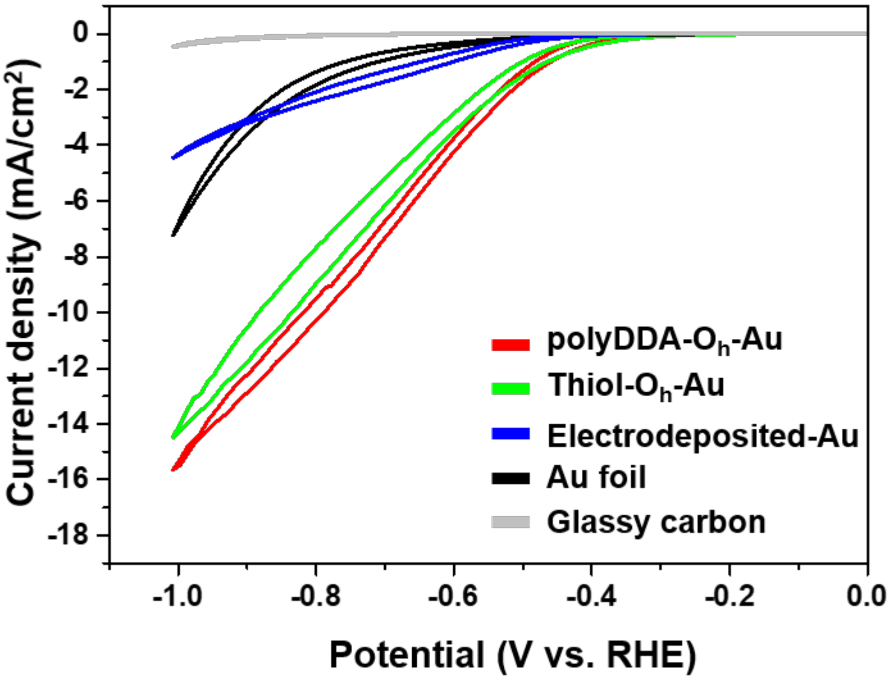

Figure S16. Cyclic voltammetry (scan rate 20 mV/s) of polyDDA-O_h-Au, Thiol-O_h-Au, Electrodeposited-Au, Au foil and glassy carbon electrode in CO_2 saturated 0.1 M $KHCO_3$ for comparing ligand effect on CO_2RR.

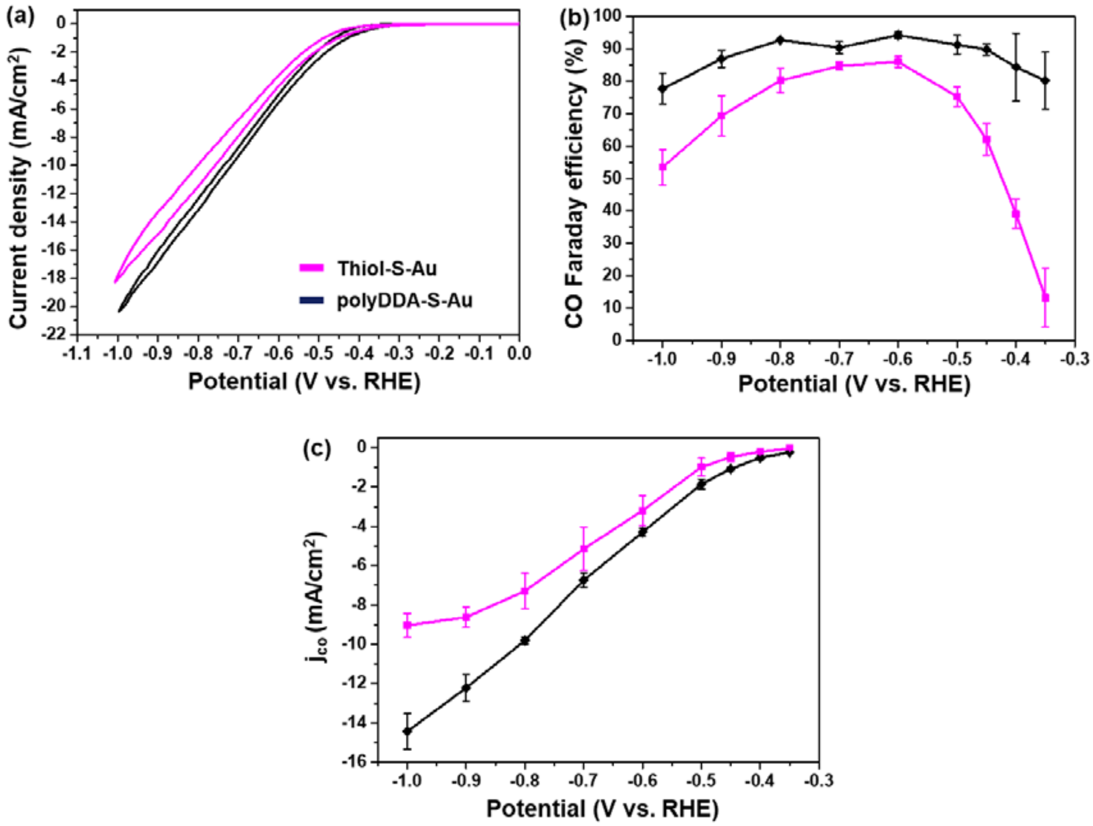

Figure S17. (a) CVs of polyDDA-S-Au, Thiol-S-Au electrodes in CO_2 saturated 0.1 M $KHCO_3$ for comparing ligand effect on CO_2RR. (b) Faraday efficiency of CO, and (c) Partial current density of CO generated by polyDDA-S-Au, Thiol-S-Au electrodes.

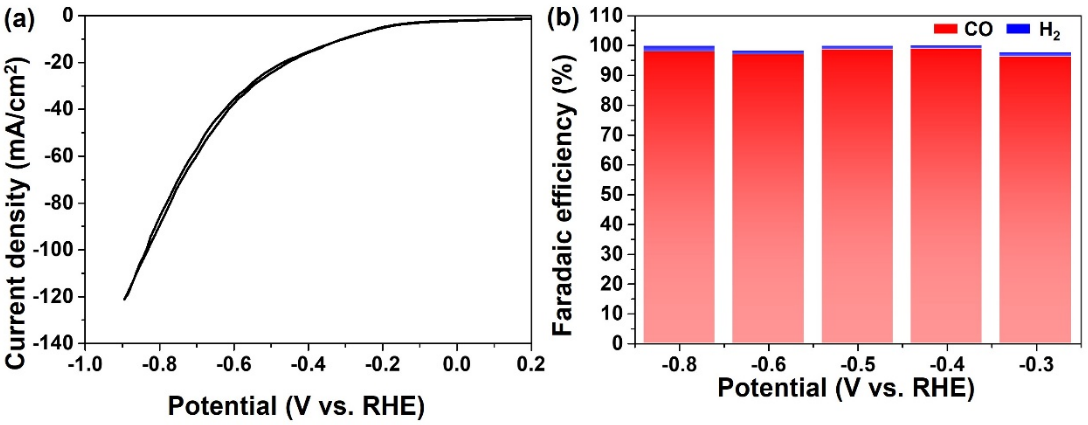

Figure S18. (a) Cyclic voltammetry (scan rate 20 mV/s) of polyDDA-O_h-Au on GDE in CO_2 saturated 1M KOH, and (b) Faradaic efficiency (blue: H_2 and red: CO_2) depending on applied potentials.

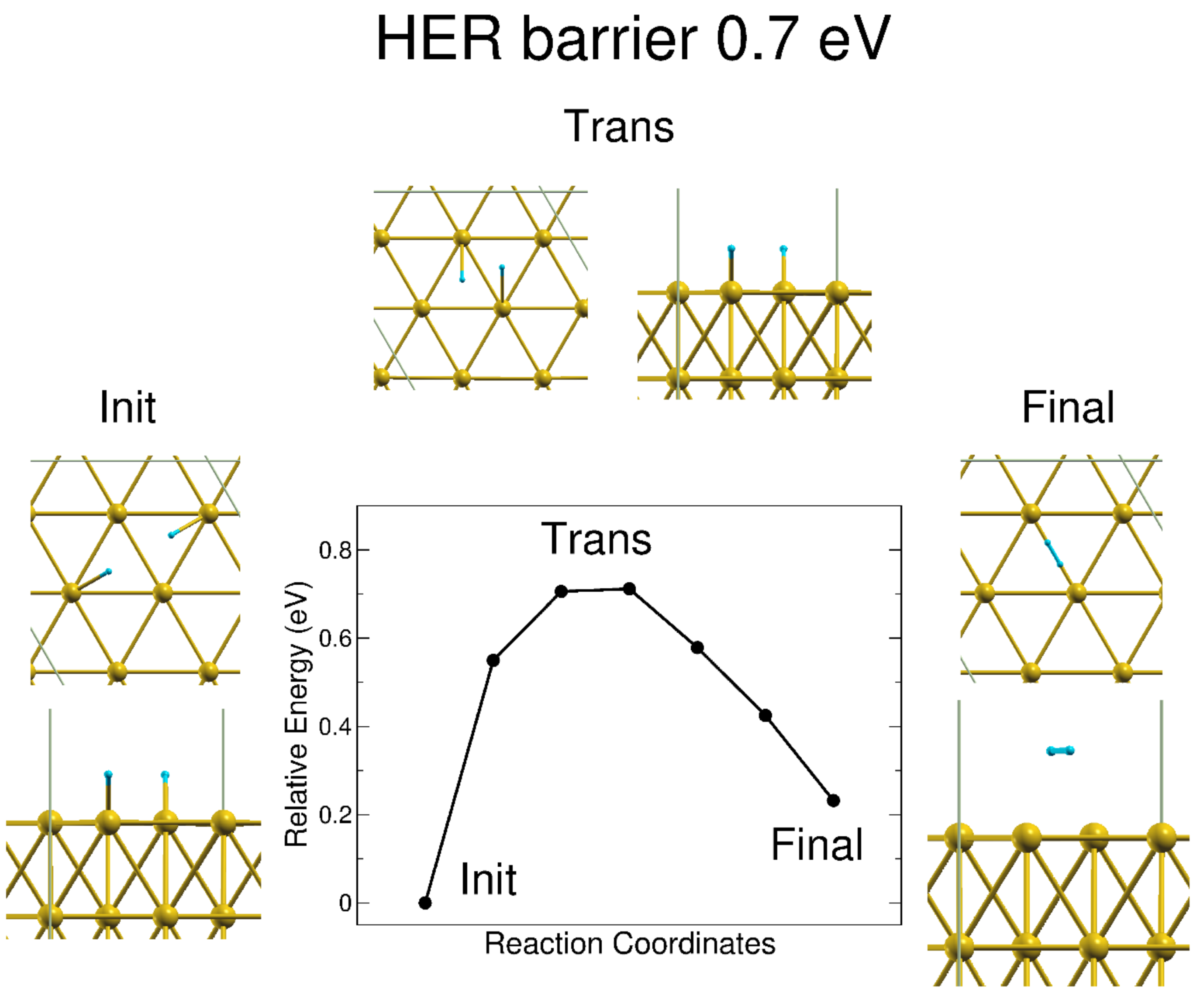

Figure S19. NEB calculation results for HER without surface Cl atoms on Au (111) surface. Top and side views of each structure are shown in upper and lower positions. Yellow and blue balls represent Au and H atoms, respectively.

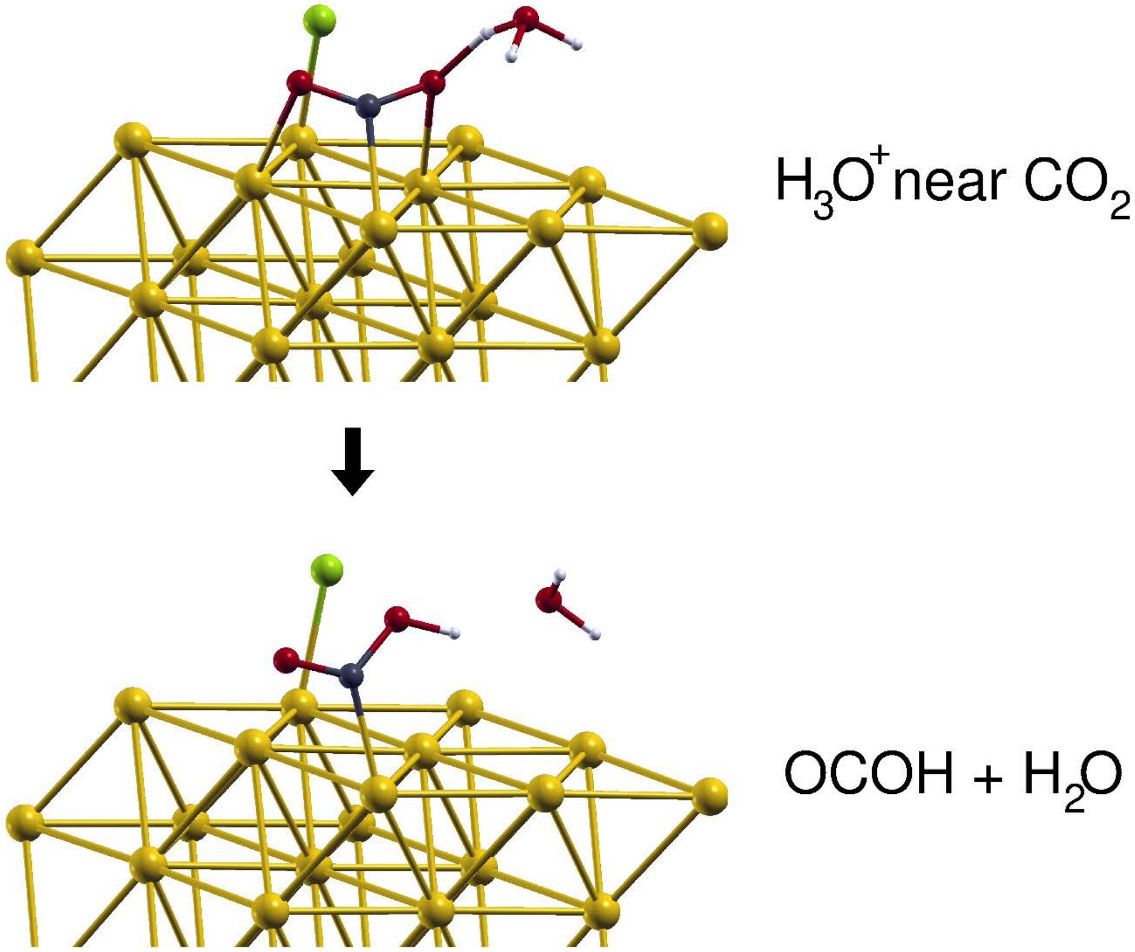

Figure S20. H_3O^+ approach to CO_2 and OCOH+H_2O formation process from DFT calculations with surface Cl atoms.

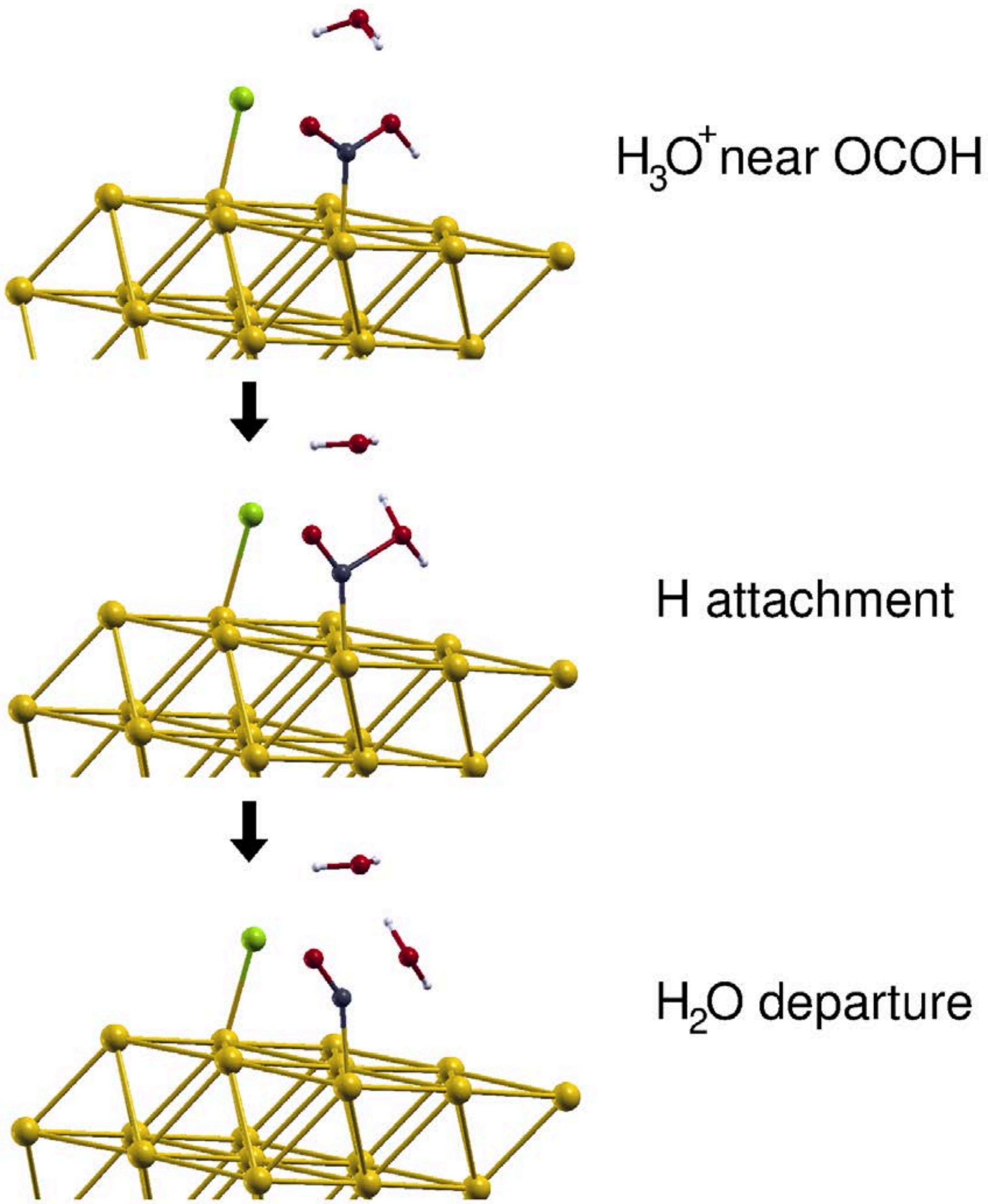

Figure S21. H_3O^+ approach to COOH and CO + H_2O formation process from DFT calculations with surface Cl atoms.

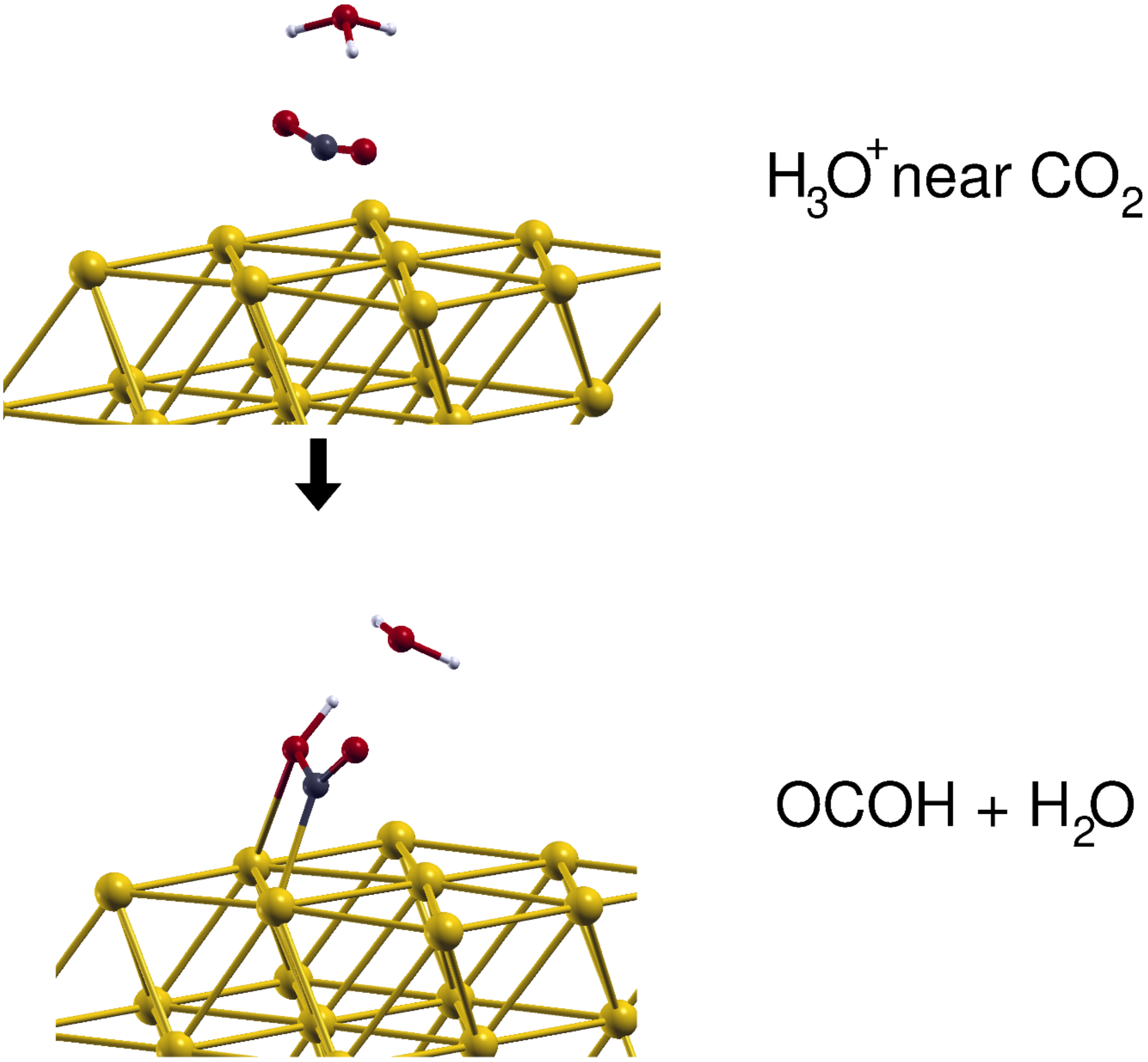

Figure S22. H_3O^+ approach to CO_2 and OCOH + H_2O formation process from DFT calculations without surface Cl atoms.

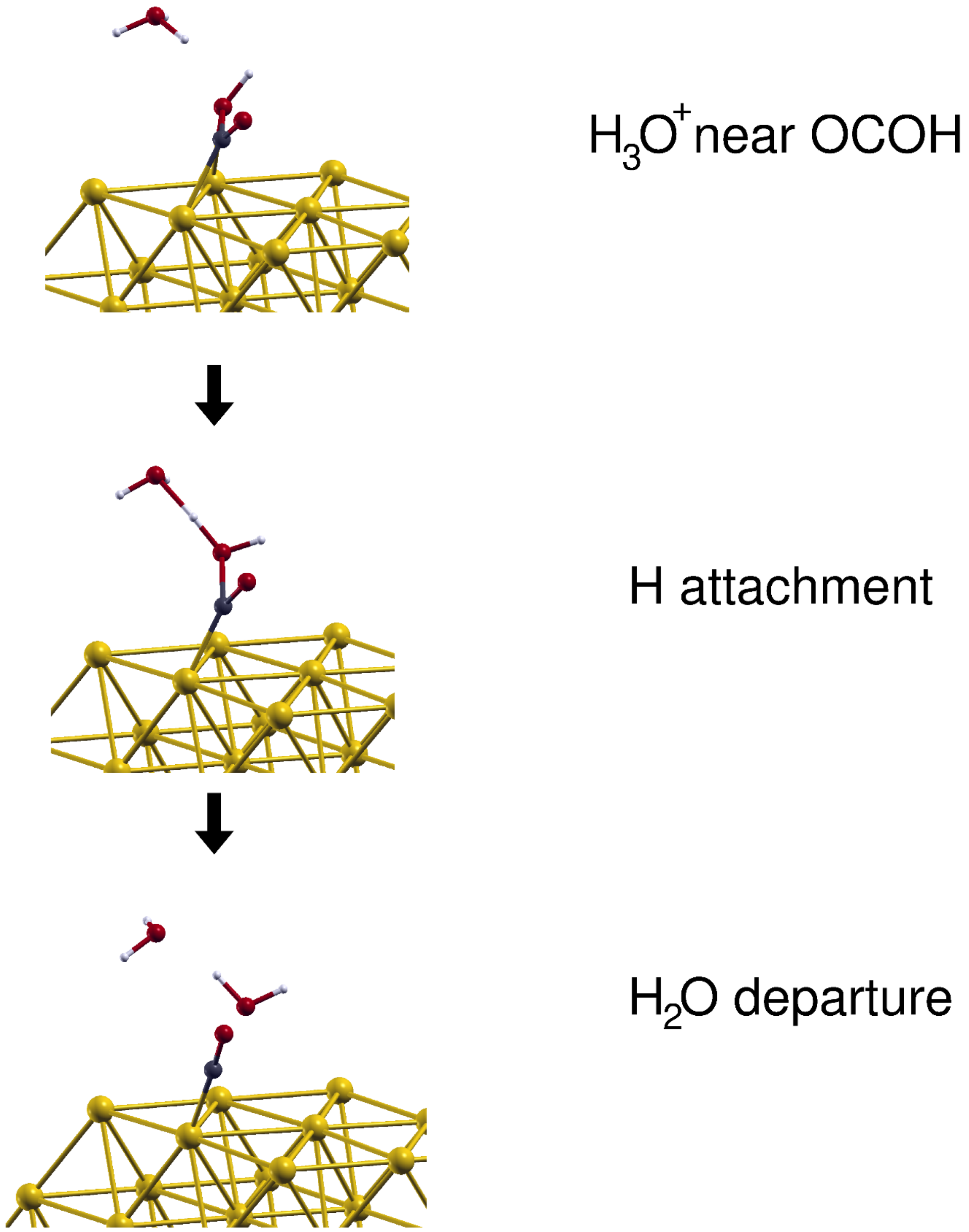

Figure S23. H_3O^+ approach to COOH and CO + H_2O formation process from DFT calculations without surface Cl atoms.

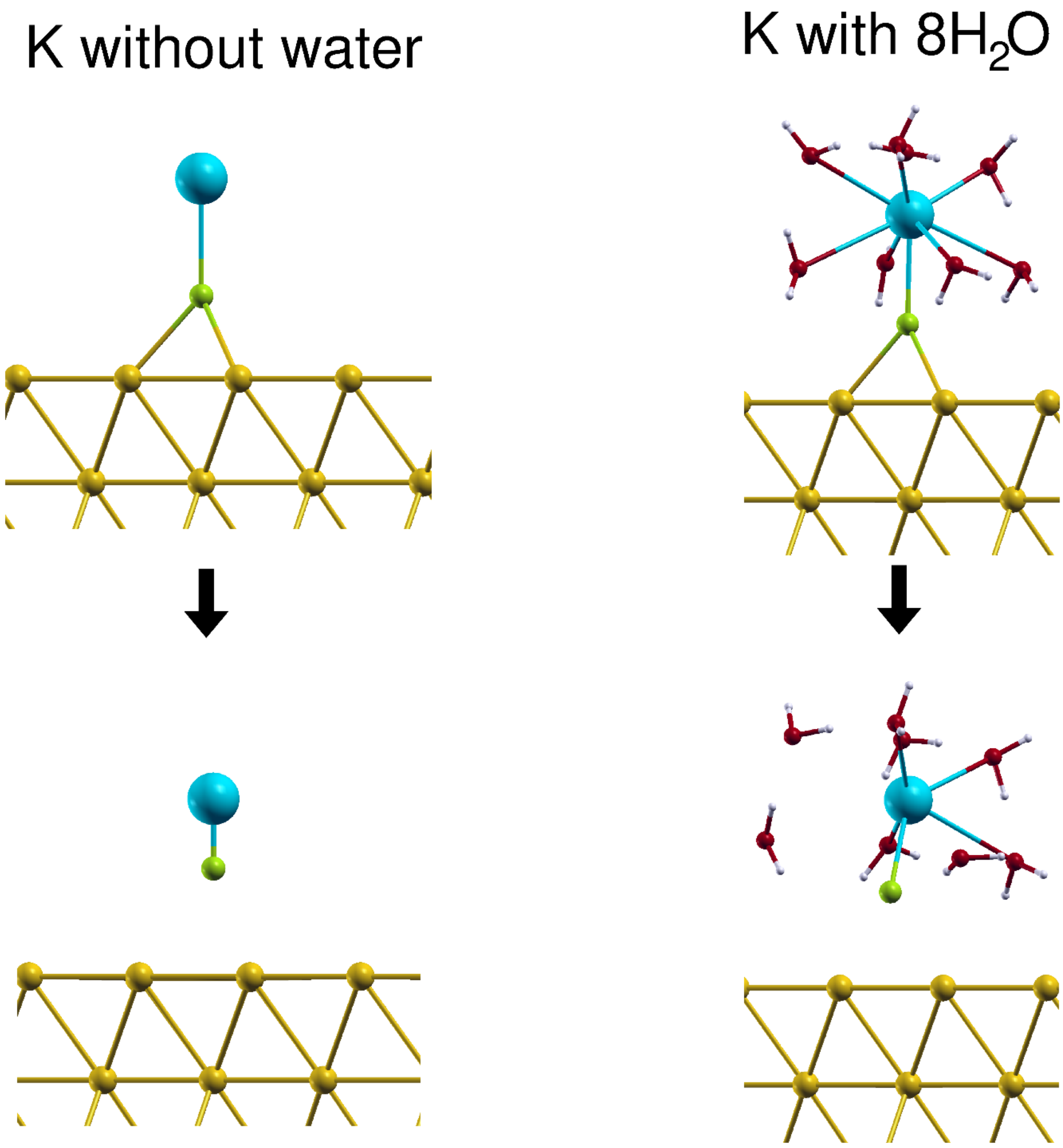

Figure S24. Vertical approach of K atom to surface Cl with and without water molecules. Blue, green, yellow, red and white balls represent K, Cl, Au, O and H atoms, respectively.

	Zeta-potential (mV)	STDEV (mV)
**polyDDA-O_h-Au**	**+57.49**	**9.66**
**polyDDA-TO-Au**	**+42.43**	**7.63**
**polyDDA-S-Au**	**+33.34**	**4.18**

Figure S25. Zeta potential of polyDDA-Au samples.

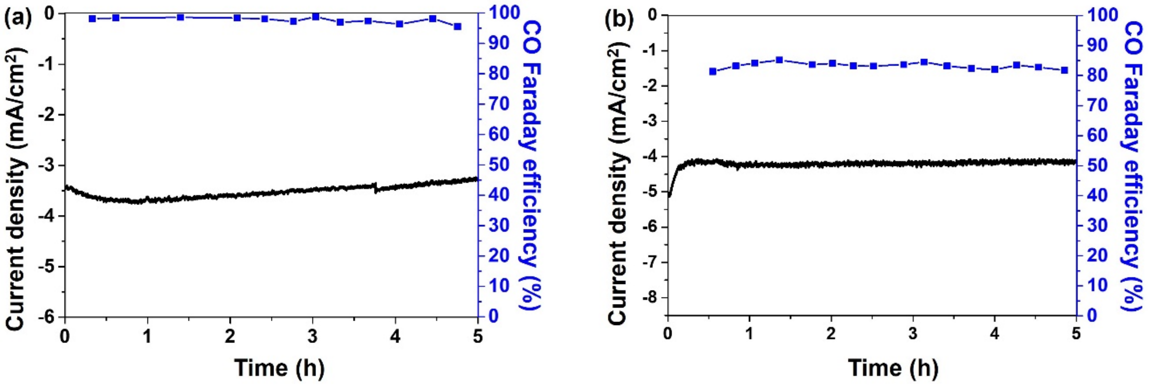

Figure S26. Chronoamperometric measurement of (a) polyDDA-Ag at an applied potential of -0.8 V (vs RHE), and (b) polyDDA-Zn at an applied potential of -0.9 V (vs. RHE) for 5 h.

Refernece

[1] G. Kresse, D. Joubert, *Phys. Rev. B* **1999**, *59*, 1758-1775.

[2] G. Kresse, J. Furthmuller, *Phys. Rev. B* **1996**, *54*, 11169-11186.

[3] J. P. Perdew, K. Burke, M. Ernzerhof, *Phys. Rev. Lett.* **1996**, *77*, 3865-3868.
